# Impact of Capital Adequacy on the Profitability of Microfinance Institutions in Nepal

**DOI:** 10.12688/f1000research.178815.1

**Published:** 2026-03-27

**Authors:** Basu Dev Lamichhane, Satyanarayan Choudhary, Bharat Ram Dhungana, Manika Shrestha

**Affiliations:** 1 Saraswati Multiple Campus, Tribhuvan University, Kathmandu, Nepal; 2 School of Business, Pokhara University, Pokhara, Nepal; 3 Freelance Researcher, Kathmandu, Nepal

**Keywords:** Capital adequacy, MFIs, profitability, regulatory institution, sustainability

## Abstract

**Background:**

Capital adequacy is essential for the sustainable operation of microfinance institutions (MFIs). MFIs can expand their outreach to the low-income and marginalized people if the institutions are sustainable. Maintaining adequate capital is important from a regulatory perspective to ensure financial stability and sustainability. The objectives of this study are to examine the impact of capital adequacy on the profitability of Nepalese microfinance institutions (NMFIs).

**Methods:**

This study is based on secondary data collected from the annual reports of the selected microfinance institutions. A descriptive and causal comparative research design was used. The study sampled 10 microfinance institutions from 57 purposively. The independent variables were core capital ratio, supplementary capital ratio, cash reserve ratio, capital adequacy ratio, and leverage ratio. The dependent variables were Return on Assets (ROA) and Return on Equity (ROE). The purposive sampling method was used.

**Results:**

The findings of the study revealed that all relationships were positive, except for the leverage ratio, which had a negative relationship with all other variables. The study variables CCR, SCR, CAR, CRR, and LR significantly affect ROA, except LR. Similarly, the dependent variable ROE also has significant impacts on the study variables CCR, SCR, CAR, and CRR, whereas the LR has an insignificant impact on the leverage ratio.

**Conclusion:**

The study brings real acuities to light, as intended by proper executives and governments, leading to a robust economic recovery and ensuring sustainable growth in Nepalese microfinance institutions. Regulators and policymakers may focus on improving the financial and non-financial performance of MFIs, including expanding their outreach to marginalized and rural populations through monetary policy.

## 1. Introduction

MFIs play an important role in influencing the capital adequacy of financial institutions, particularly in cutting-edge emerging economies, by providing imperfect access to banking services ( Anggraini & Suputra, 2021). The aptitude of MFIs to preserve a strong Capital Adequacy Ratio (CAR) remains perilous, aimed at their economic durability, besides flexibility alongside credit and operational threats originate that supervisory outlines and capital structure meaningfully influence the CAR of MFIs, as institutions dependent on allowances and easy-going loans regularly express effort in supporting adequate capital barriers ( Abate et al., 2014).

CAR plays a vital role in confirming the financial permanency and durability of MFIs, especially in fostering financial prudence. Profitability, loan portfolio quality, and official size are identified as important elements of CAR amongst MFIs ( Bata et al., 2021).

The profitability amplified interior capital growth, thus establishment CAR in Sub-Saharan African MFIs. MFIs, through expanded capital structures, mainly those relying on engaged earnings and equity, remain better placed to preserve advanced CAR intensities and survive economic astonishments ( Bogan, 2012).

Though the mission-driven MFIs frequently face a trade-off between community outcomes and economic durability. MFIs are inclined to lend to weaker CARs due to lenient lending practices and to the need to generate compact interest income ( Tchakoute-Tchuigoua, 2010). Accommodating, MFIs also regularly relax capital adequacy requirements in the service of community purposes, although shareholder-owned MFIs usually preserve a durable CAR, subject to severe monetary supervision and governing acquiescence. Together, these results indicate that MFIs’ contribution to economic presence was meaningful and that their ability to maintain adequate capital levels is shaped by official alignment, profitability, supervisory requirements, and operational effectiveness ( Bei et al., 2025).

Capital adequacy is an important support for economic stability in the banking and microfinance sectors. It states that economic institutions must maintain a minimum level of capital, proportional to their risk-weighted assets, to protect shareholders and preserve complete certainty ( Humta et al., 2024). The CAR is also familiar through the Capital to Risk-Weighted Assets Ratio (CRAR), a key supervisory tool used globally to assess a group’s or microfinance institution’s ability to attract new customers and remain healthy during times of economic pressure ( Nguyen, 2020). CAR consists of two main components: Tier 1 capital, which includes essential equity and disclosed capital, and Tier 2 capital, which includes subordinated debt and mixed instruments, contributing to defense in insolvency situations ( Shabani et al., 2019).

The need to preserve adequate capital is emphasized in frequent supervisory guidance, including the Basel accords, international standards for economic sector regulation. Institutions, through durable CAR, are considered extra resilient to credit, market, and operational risks, thus playing a crucial role in fostering confidence between investors and depositors. Capital assistance not only serves as a barrier against financial surprises but also as an instrument for aligning executive risk incentives and signaling solvency to creditors and market participants ( Benink & Wihlborg, 2002).

MFIs focused their specific expression, such as exclusive capital adequacy trials, owing to their operational emphasis on underserved, low-income inhabitants. These institutes operate in risk-prone environments due to limited access to inferior capital markets and are therefore additionally susceptible to loan evasions, liquidity deficits, and operational fiascos ( Jadhav et al., 2021). Regulatory institutions such as the Nepal Rastra Bank adopt approved capital adequacy norms tailored for MFIs to improve financial inclusion while reducing total risk. CAR necessities in Nepalese MFIs are important not only for obedience, but also for operational flexibility and investor confidence ( Goet, 2022).

Microfinance institutions play a vital role in poverty alleviation and broader financial development ( Lamichhane, 2020). However, their longstanding toughness centers on the capital organization’s overall performance. Capital indicators of abuse of depositor capital, intensifying financial vulnerabilities, and corroding community belief ( Shrestha, 2022). Capital adequacy confirms that institutions align their communal assignment with economic foresight, thereby safeguarding depositor benefits and preserving financial system integrity. Capital adequacy is not merely a practical prerequisite; it is also a calculated characteristic of banking that affects supervisory decisions, risk tolerance, and shareholder performance ( Mainelli, 2004).

The durable capital adequacy outline remains crucial for the feasibility and reliability of microfinance institutions, particularly in evolving markets. It allows the institutes to assume supervisory responsibilities, cushion against adverse tremors, and advance their education for the future ( Kunjeda, 2024).

The CAR remains an important indicator of a financial institution’s stability and flexibility, measuring its capital adequacy relative to its risk-weighted assets. Although usually viewed through a supervisory acquiescence lens, there is a cumulative credit to the lively association between a bank or microfinance institution’s effectiveness and its capital adequacy. Viability, unhurried, complete indicators such as ROA, ROE, and net profit margin (NPM) can knowingly affect CAR by increasing capital and reducing the need for external subsidy ( Indriani et al., 2020).

Experiential indication reliably underscores that advanced profitability underwrites a sturdier capital dishonorable. Profitable institutions are better positioned to recall earnings, which then underwrite Tier 1 capital indispensable for conference CAR requirements ( Shabani et al., 2019). Earnings, as a means of achieving profitability, remained a primary element of CAR in microfinance institutions, particularly in settings with imperfect access to equity or subordinated debt ( Bogan, 2012). Microfinance institutions, through consistent profitability, exhibited high CAR levels, thereby supporting the sequence between capital adequacy and stability ( Jadhav et al., 2021).

CAR is frequently careful and an effort to profitability; the opposite association, where profitability is obsessed with capital reserves, has important procedural implications, predominantly aimed at smaller banks and MFIs ( Mamun et al., 2022). Although preserving CAR upstairs supervisory beginnings, failures in profitability finished time negatively impacted the accretion of core capital, consequently weakening long-term capital adequacy. This advises that effectiveness is not only a presentation metric but also a calculated asset that provisions compliance and toughness ( Chalise, 2023).

Tier 1 capital’s optimistic tone complements net profit in Nepalese commercial banks, reinforcing the belief that core principal fundamentals are strengthened, resulting in consistent earnings ( Goet, 2022). Profitability definitely predisposed capital relations by empowering institutions to increase capital buffers without experiencing new liabilities. The toughness of such capital constructions, particularly in microfinance institutions, is contingent on their capability to produce stable revenues ( Adjei et al., 2020).

Profitability-related unpredictability effects CAR in Iranian banks. Their schoolwork determined that income inconsistency is relatively more significant than dependable effectiveness indicators to variability in capital planning. Capital adequacy seeks to avoid impairing the level; nonetheless, the advantage and toughness of profitability ( Taherinia & Baqeri, 2018).

There are positive associations between profitability (especially ROE and NPM) and CAR among Nepalese banks, indicating that although regulatory requirements were met and long-term capital assets were maintained, these banks yielded low returns. Liquidity and credit quality matter considerably in determining the profitability of CAR connotation, primarily in organizations in industries with high population density ( Bata et al., 2021).

Profitability attracts the entire risk group and capital growth, as profitable institutions exhibit less reluctance to increase capital, weaken equity, or take on luxurious debt. Effectiveness, in turn, influences CAR to improve loan volume and operational efficiency, thereby reducing risk-weighted asset development ( Indriani et al., 2020).

Lastly, supervisory standpoints recognize this suggestion in the minimum CAR ethics. Basel III developments encourage revenue conservation by requiring counter-cyclical principal buffers, suggesting that the necessity of efficiency serves as the primary driver of conflict in capital preservation ( Jadhav et al., 2021). Profitability-driven principal expansion is imperative for stability in immature financial schemes. Profitability portrays an energetic, multi-dimensional character in its impact on the Capital Adequacy Ratio. It increases a monetary institution’s capability to build internal capital cushions, reduce dependence on external capital, and fulfill through controlling values. The interdependence of profitability and CAR is particularly pronounced in microfinance and emerging nation settings, where capital markets are underdeveloped and regulatory settings are evolving ( Shabani et al., 2019).

In Nepal, Laghubittiya Bittiya Sanstha Limited (LBSL) states that Microfinance Financial Institutions Limited is required to maintain a minimum CAR of 11% to safeguard monetary stability and protect the interests of depositors against investors. Preserving these typical chains, like both recognized toughness, broader monetary addition efforts. For this research, 10 important LBSLs were decisively selected based on their strong operational measures, sound economic records, and extensive topographic coverage. These institutions include Chhimek Laghubittiya Bittiya Sanstha Limited (CLBSL), Nirdhan Utthan Laghubittiya Bittiya Sanstha Limited (NULBSL), Forward Laghubittiya Bittiya Sanstha Limited (FLBSL), First Microfinance Laghubittiya Bittiya Sanstha Limited (FMLBSL), Grameen Bikash Laghubittiya Bittiya Sanstha Limited (GBLBSL), Mero Microfinance Laghubittiya Bittiya Sanstha Limited (MMLBSL), Sana Kisan Bikas Laghubittiya Bittiya Sanstha Limited (SKBLBSL), RSDC Laghubittiya Bittiya Sanstha Limited (RLBSL), NMB Laghubittiya Bittiya Sanstha Limited (NMBLBSL), and NADEP Laghubittiya Bittiya Sanstha Limited (NADEPLBSL). These LBSLs characterize a consistent example for exploring the influence of capital adequacy on effectiveness within Nepalese microfinance institutions.

### 1.1 Problem statement

MFIs in Nepal play a significant role in advancing financial inclusion by providing credit and other financial services to underserved individuals, predominantly in rural and low-income areas. Though the toughness and economic well-being of these organizations are contingent heavily on their capital structure and profitability. The Capital Adequacy Ratio (CAR), which measures the extent to which an institution’s capital exceeds its risk-weighted assets, is an important indicator of an institution’s flexibility and risk-bearing capacity ( Nguyen, 2020). Central banks, such as the Nepal Rastra Bank (NRB), set the lowest CAR norms to ensure that MFIs are adequately capitalized, thereby mitigating potential economic shockwaves and avoiding liquidation ( Kunjeda, 2024). Though these necessities are indispensable for preserving financial stability, the impact of capital adequacy on the profitability of MFIs remains a compound and frequently discussed problem.

Numerous studies indicate that capital adequacy can comprise both positive and negative elements, depending on profitability. Conserving the developed capital obstructions might reduce the risk of escape, improve dependability, and recover backup situations, thereby being instrumental to profitability ( Shabani et al., 2019). Irrationally close were tall capital phases might hinder lending ability, limit income clustering, and decrease return on equity, predominantly in capital-scarce locations like Nepal ( Mamun et al., 2022). These varying results generate indecisiveness among policymakers and advisers. Furthermore, the Nepalese microfinance segment views the whole thing in excellent circumstances, including high operating costs, small loan sizes, and unprotected borrower groups, which can change the traditional model among CAR and profitability, tacit in commercial banks or large financial institutions ( Adjei et al., 2020).

Predominantly, the nonfiction in Nepal has focused on the impact of capital adequacy on the commercial banking sector, leaving a significant gap in investigating MFIs ( Kunjeda, 2024). Dissimilar commercial banks and MFIs agree with a custom that repeatedly lacks credit antiquity, collateral, or formal employment, snowballing their experience to credit and working risks ( Muriu, 2016). Then, the applicability of capital adequacy standards to an establishment’s requirements requires more experimental inspection. In calculations, global agendas such as Basel II and Basel III are progressively shaping NRB’s governing carriage, raising the reputation of empathetic effectiveness cooperates through the capital requirements under these evolving circumstances ( Jadhav et al., 2021).

It is essential to assess whether profitability underwrites the development of CAR through firming interior capital assets, or if capital necessities themselves overwhelm profitability due to preventive financial illiquidity. Deprived of lucidity on this relationship, controllers might implement capital strategies that inadvertently challenge institutional recital, or MFIs can undercapitalize in search of immediate profits, cumulative the risk of universal disappointment. This study aims to explore the impact of profitability on the Capital Adequacy Ratio of Nepalese microfinance institutions, highlighting the connection to the current research gap and delivering criminal visions for controllers, policymakers, and privileged officials ( Chalise, 2023).

This study examined the intricate association between capital adequacy and profitability in Nepalese microfinance institutions (MFIs). There were mainly cutting-edge supervisory situations that stress financial stability, though the institutions endeavor to carefully attend to vulnerable inhabitants. Preserving CAR from top to toe remains important for jeopardy fascination and long-term resilience, but it can also compel the provision of MFIs, thereby affecting their success ( Goet, 2022). As an academic, it was attentive to discovering the controlling necessities, such as CAR influencing the economic performance of MFIs, especially after these institutions’ appearance, leading to acceptance values, while also bringing socio-economic impact ( Kunjeda, 2024). Moreover, the limited leverage due to high capital requirements can constrain MFIs’ ability to offer competitive loan products and reduce their focus on high-return lending, further affecting profitability ( Jadhav et al., 2021). Understanding this dynamic is crucial for aligning financial regulation with the growth and durability of MFIs in Nepal.

Given the importance of capital adequacy in microfinance institutions, it has been necessary to study various aspects, the impact, and the status of the capital adequacy norms and standards in the context of Nepal. Various questions arise about the subject. So the specific statements of the problems of this study will be as follows:

i. What is the current status of the capital adequacy ratio, core capital ratio, supplementary capital ratio, cash reserve ratio, leverage ratio on profitability, return on assets, and return on equity ratio?
ii. What is the relationship between the core capital ratio, supplementary capital ratio, capital adequacy ratio, cash reserve ratio, leverage ratio, return on assets, and return on equity ratio?
iii. What is the impact of the variables, capital adequacy ratio, core capital ratio, supplementary capital ratio, cash reserve ratio, leverage ratio, on profitability, like return on assets and return on equity ratio?

## 2. Objectives of the study

The general objective of this study is to evaluate the impact of capital adequacy ratios on the profitability of microfinance institutions in Nepal. The specific objectives of the study are:

i. To assess the current status of capital adequacy ratios, such as core capital ratio, supplementary capital ratio, cash reserve ratio, and leverage ratio, on profitability, such as return on assets and return on equity ratio.
ii. To analyze the relationship between the core capital ratio, supplementary capital ratio, capital adequacy ratio, cash reserve ratio, leverage ratio, return on assets, and return on equity ratio.
iii. To examine the impact of the variables, capital adequacy ratios, such as core capital ratio, supplementary capital ratio, cash reserve ratio, leverage ratio, on profitability, such as return on assets and return on equity ratio.

## 3. Literature review

A literature review is a structured summary of existing research and theories related to a specific topic, capital adequacy, and its impact on the profitability of microfinance companies in Nepal ( Chalise, 2023). It typically includes a theoretical review, which discusses relevant theories explaining key concepts and relationships; an empirical review, which examines findings from previous studies based on real-world data; and identifies research gaps, highlighting areas where evidence is lacking or inconsistent. This research examined models, such as capital structure theory and the risk-return trade-off, that explain the effects on capital adequacy and profitability. Empirical revisions examined the diverse consequences of this connection in Nepalese microfinance institutions. However, research on capital adequacy and its impact on the profitability of Microfinance institutions remains limited.

### 3.1 Theoretical review

A theoretical review summarizes and elucidates the main concepts associated with a revision’s issue, as well as providing a basis for understanding the variables and their relations. In the context of capital adequacy, in addition to the profitability of microfinance institutions.

***3.1.1 Modern Portfolio Theory (MPT)***

Modern portfolio theory (MPT) was established by Markowitz in 1952. That is founded on the impression that a stockholder canister concept an “effective” selection that exploits probable reappearance aimed at an assumed equal of risk. This model is primarily applicable to MFIs in Nepal, as it emphasizes expanding economic assets to mitigate risk. Intended for MFIs, preserving a CAR remains important for managing risks, including credit, liquidity, and operational risks, which are characteristic of their occupational performance. A developed CAR allows MFIs to cover unforeseen fatalities, such as evasions on microloans, without exposing the institution’s financial stability. This is the chance to help realize the institute’s ideal risk-adjusted revenues, ensuring it can continue to offer facilities while preserving effectiveness.

MPT advises that MFIs would expand their credit selections and secure adequate principal to cushion against probable fatalities, therefore certifying that their success is not jeopardized by extreme experience to risk. By conserving the best equilibrium between risk and principal, MFIs can protect themselves from the inconsistency of economic surprises while maintaining earnings and earning low-cost returns. This theory provides the knowledge that a well-calculated CAR container facilitates key acts essential to long-term stability and dependable effectiveness.

***3.1.2 Risk-Based Capital Adequacy Framework (RCAF)***

The risk-based capital adequacy background, established by the Basel Committee on Banking Supervision in 1999, highlights the essential aim of economic institutions to preserve capital in balance with the risks they accept, specifically credit, market, and operational risks. The outline’s request is vital for Nepalese MFIs, as they tend to operate in environments where the risk of mortgagor avoidance arises due to financial variability or imperfect access to economic capital. By guaranteeing that MFIs preserve a satisfactory CAR, they are better prepared to survive economic pressure. This organization analyzes the essential capital created on the institute’s risk-weighted assets to assist in determining the minimum capital required to absorb potential losses. Aimed at MFIs, this supervisory method not only safeguards the individual safeguards they encounter, but also improves their capability to provide reasonable acknowledgment to underserved inhabitants. Additionally, it delivers protection against monetary tremors, averting liquidation and protecting investors’ funds. Upholding a suitable CAR within this outline permits MFIs to meet supervisory norms while ensuring stability among investors ( Basel Committee on Banking Supervision, 2010). It also confirms that these institutes continue to generate revenue through actionable intelligence to mitigate risks efficiently, which is important for continuous development and improved consistency in cutting-edge microfinance.

***3.1.3 The Agency Theory (TAT)***

Agency theory was advanced by Michael Jensen and William Meckling in 1976 to examine the relationship between principals (owners) and agents (managers) within a society, particularly regarding how the benefits of these two groups may not always align. In the case of MFIs, the model advises that executives may remain incentivized to take extreme risks or engage in activities that benefit them individually, even when they are not in the best interests of the institution’s owners or shareholders. This can undermine the group’s consistency and effectiveness. A resilient CAR canister can help with this by ensuring MFIs maintain adequate equity capital to attract potential borrowers, thereby reducing the need for risky liability support. Through preserving an adequate CAR, directors are less likely to incur extreme risks, as the economic defense protects the institution and its investors after potential economic catastrophes. This reduces the likelihood of conflict among executives and proprietors, as joint gatherings are incentivized to preserve the institution’s financial health. A vigorous CAR so that theaters a character in bringing to the forefront the benefits of managers and proprietors, encouraging practical decision-making and long-term effectiveness. By preserving the institute amid economic variability, the CAR nurtures an additional helpful and enduring association across all complex gatherings, refining together working efficiency and overall success.

### 3.2 Empirical review

An empirical review is important because it offers evidence-based insights by examining actual data and results from earlier studies. It supports authorized or task-theoretical norms, exposes designs or relations between variables, and highlights practical results in similar settings.

Bei et al. (2025) assessed the impact of CAR on banks’ economic performance in China. It was principally in a well-lit environment of swelling FinTech amalgamation. There was expanding panel data from 2013 to 2023, covering 25 banks, besides retaining fixed-effects reversion. They measured CAR’s possessions continuously, as well as ROA, ROE, and interest margin (NIM). This study found that the reinforced CAR moderately buttressed efficacy improvements after FinTech, with risk flexibility and provisions improving even productivity. The research experimentally shows a reduction in nearby returns at higher capital stages. The researcher achieved that banks would preserve adequate capital cushions through leveraging technology-driven proficiency to improve the performance in developing monitoring environments.

Ikpesu and Oke (2025) analyzed the impact of CAR on bank effectiveness among Nigerian commercial banks. The results provided valuable insights into the association’s worldwide vision. The research confirmed that, though CAR remains important for confirming financial stability and preserving banks against credit risks. There were also enforced limitations on profitability through restrictions on banks’ lending capacity and increases in operating expenses. The GMM estimation system was applied to panel data on 12 major Nigerian banks from 2010 to 2019. It was found that there was a significant negative association between CAR and return on assets (ROA). It was portentous that firmer capital requirements tend to reduce banks’ earnings. The results achieve the essential aim of a composed regulatory outline that confirms satisfactory capital buffers without discouraging bank profitability and market attractiveness.

Johnson et al. (2025) examined the impact of CAR, stratified by U.S. bank profitability, over the period 2015 to 2024. There was a spread over fixed-effects panel regression analysis. They indicate that developed CAR commonly recovers banks’ flexibility to economic tremors and positively affects profitability metrics like return on assets (ROA) and NIM. Though this research illustrates a lessening return effect, where disproportionately in height CAR heights it might oblige lending capacity, leading to abridged profitability over the period. The researcher concluded that U.S. banks’ essential balance-monitoring capital requests could be met through tactical capital management, thereby improving both financial stability and effectiveness.

Liza (2025) evaluated the impact of capital adequacy, in addition to asset quality, on bank performance in Bangladesh. It was intensive on profitability metrics such as return on assets (ROA), operating income, and CAPE. This study is intended to analyze the association among capital adequacy, asset quality, and bank profitability; examine essential changes in these relationships across the pre- and post-Basel supervisory epochs; and offer strategy insights to support banking-sector consistency. This research is conducting a multi-method, measurable investigation using panel data from 2011 to 2023. Fixed-effects regression simulations, ordinary least squares (OLS), and virtual exams remained pragmatic to investigate data collected from annual reports of Bangladesh-based banks and macroeconomic indicators from the Bangladesh Bank. It was found that company size, movement mix, cost organization, curiosity rate, GDP growth rate, asset quality, and net interest margin absolutely affect profitability, although capital adequacy and inflation rate adversely affect it. This research acknowledged important modifications in profitability subtleties post-Basel application, observing that profitability decreased as capital adequacy ratios increased, whereas asset quality’s positive effect on profitability was supported. It was concluded that although preserving capital adequacy is vital for risk management, excessive regulatory capital requirements may diminish profitability. Firming up supremacy and directing asset-quality development are important for maintaining a sustainable bank presence in Bangladesh.

Thapa et al. (2025) examined the aspects affecting capital adequacy ratios in Nepalese commercial banks. It was focused on the company size, profitability, and recognition risk. This research found that superior banks with higher profitability tend to maintain higher capital adequacy ratios. Conversely, improved non-performing loans and increased dividend payouts significantly impeded capital adequacy ratios, indicating that banks’ essential balance profitability with practical risk management to preserve financial stability.

Warasto and Janudin (2025) analyzed the effect of the CAR on PT Bank Raya Indonesia Tbk. ‘s financial performance. There was an application of data after the series was inspected: yearly economic intelligence from 2017 to 2023. The research used multiple linear regression analysis. It was revealed that a complex CAR significantly improves the bank’s financial presentation, underscoring the importance of maintaining robust capital safeguards to ensure stability and profitability. This study proposes that operative capital management is critical for banks to achieve sustainable financial outcomes.

Humta et al. (2024) assessed the correlation between capital adequacy and bank profitability. There was an exploratory study of the regulatory effects of macroeconomic variables such as increases in interest rates and exchange rates. This research used panel data from 2010 to 2021, focusing on the banking sectors in Malaysia, Indonesia, and Turkey. The regression analysis was employed to determine how both internal and external factors prejudiced profitability. The outcomes indicated that capital adequacy, exchange rates, and interest rates have a positive influence on profitability, but inflation has an important, undesirable effect. Moreover, this research increases interest rates, thereby qualifying the association between capital adequacy and profitability; nonetheless, the interface relationships remained statistically insignificant.

Kunjeda (2024) investigated how capital adequacy affects the profitability of particular commercial banks in Nepal. There were counting together, direction, and confidentially retained institutions. This research focuses on ratios such as CAR, ROA, ROE, and NPM. There was a need to consume secondary data from financial statements. Statistical methods, such as correlation and regression analysis, were used to analyze the data. This study found that, though the banks encountered the least capital adequacy requirements as usual through the Nepal Rastra Bank. Their profitability has been decreasing over the past few years. The association between capital adequacy and ROE/NPM remained inadequately positive, although the aforementioned correlation with ROA was uncertainly destructive, and reversion analysis long-established no important influence of CAR on profitability.

Thapa (2024) examined the influence of CAR arranged by bank performance. They used panel data for 11 main groups, covering the financial years 2013/14 to 2022/23. This research actively correlates with regression analysis to assess just how CAR, laterally through Tier I and Tier II capital, non-performing loans (NPL), liquidity, and total deposit levels. It was subjective profit measures like return on assets (ROA), in addition to earnings per share (EPS). These consequences showed that total CAR and Tier I capital significantly affect ROA and EPS. It was representative that stronger core capital improves profitability. Conversely, Tier II capital and higher credit capacities negatively obstructed the profitability. Moreover, higher NPL ratios were associated with lower ROA, whereas improved liquidity was associated with higher ROA and EPS. These results highlight that, in the Nepalese context, preserving vigorous core capital, along with management asset quality, is precarious for exploiting bank effectiveness.

Chalise (2023) analyzed the impact of capital adequacy on the profitability of Nepalese commercial banks over a ten-year period from 2013 to 2022. This study used secondary data from 20 commercial banks that participated in a rummage sale to examine key economic indicators such as CAR, debt-to-equity ratio, advances-to-assets ratio, and management refuges to total investment ratio. The answers exposed that although banks consistently maintained CAR above the supervisory requirement of 10%, there remained an inverse correlation between CAR and profitability (ROA and ROE). In the short term, the debt-equity ratio and the advances-to-assets ratio have a positive impact on profitability, whereas the non-performing loan ratio creates a negative impression. The research accentuated the professional role of management capital in recovering the set presentation.

Goet (2022) examined the effects of internal banking factors, including capital adequacy, on the profitability of three commercial banks in Nepal. This research was based on panel data comprising 21 observations and focused on metrics such as ROA, ROE, Tier 1 and Tier 2 capital, total capital, and loans and advances. A regression analysis was conducted to show that net profit was substantially positively associated with Tier 1 capital, shareholders’ equity, and loans and advances. In contrast, Tier 2 capital ensured that several important results were not expressed. The credit-deposit ratio, which has an evocative impact on ROA, though additional capital adequacy variables had a resilient effect on ROE. This research documented stronger associations between profitability and core capital rudiments than with supplementary capital.

Mamun et al. (2022) investigated the association between capital adequacy and bank performance in 20 conservative banks in Bangladesh over an 11-year period (2010 to 2020). These results applied association analysis and multivariate immovable effects reversion to examine the impact of CAR, Credit Deposit Ratio (CDR), and Cost-Income Ratio (CIR) on profitability. These results exhibited that CAR and CIR remained negatively correlated through both ROA and ROE. It signified that high capital, beyond operating costs, might diminish profitability. Conversely, CDR showed a positive association with ROE, but not with ROA. The research revealed that the operative organization of capital and working costs is precarious for a bank’s profitability.

Anggraini and Suputra (2021) investigated the effect of capital adequacy, credit risk, and liquidity continuously on the profitability of 38 banking corporations listed on the Indonesia Stock Exchange (IDX) from 2015 to 2019. Purposive sampling and regression analysis identified financial influences as the primary drivers of bank performance. The results exposed that CAR and liquidity have positive and significant impacts on profitability. Similarly, the credit risk ratio has a negative effect. It was suggested that well-capitalized banks, in addition to liquid banks, tend to be more profitable, whereas poor credit management can corrode earnings. This research provided valuable insights for stockholders and banking stakeholders to measure financial fitness and a variety of knowledgeable conclusions.

Bata et al. (2021) examined the effects of capital and liquidity on the profitability of conformists in Indonesia during temporary credit difficulties using a controlled adjustment. The example involved 40 banks listed on the IDX from 2014 to 2018. This research was based on the watered-down regression model and examined the statistics through e-views software. Results showed that capital and liquidity had an important effect on profitability. It highlighted the position of comprehensive capital structure alongside cash flow organization. Although credit difficulties are not unswervingly influenced by success. They meaningfully watered down the association between capital and profitability. But no controlling consequence was instituted amid credit difficulties with liquidity.

Jadhav et al. (2021) assessed the impact of CAR on the profitability of India’s private segment banks under Basel III standards. This research was comprised of data since the main banks comparable HDFC, ICICI, Kotak Mahindra, AXIS, and YES Bank have a five-year historical. There was a used person’s correlation in addition to the theory, which was difficult. The investigation is expected to determine whether developed CAR prejudiced bank net incomes. The answers discovered a quantifiable result of CAR on profitability. It was important that, although satisfactory capital safeguards were in place, supervisory amenable remained. It might similarly pressure income competitors’ uncertainty, which is not accomplished efficiently. It was commended calculated capital administration to continue profitability and attractiveness.

Indriani et al. (2020) analyzed the impact of CAR, in addition to Third Party Funds (TPF), on lending and ROA in community banks listed on the Indonesia Stock Exchange between 2012 and 2015. The sample company involved 15 banks, selected purposively. Panel data regression was used to analyze financial intelligence. The results specified that CAR harmfully pretentious ROA, which signifies the additional capital capacity boundary revenue-generating events such as lending. Although TPF had a positive impact on ROA, it also increased lending. These opinions on the significance of payment enrollment and the effective use of reserves are compelling in terms of their effectiveness.

Lamichhane (2020) investigated the serious character of microfinance in empowering women, mainly in the country and marginalized groups. Using an expressive investigation strategy, the training emphasizes that the microfinance courses are exclusively tailored to meet the needs of unfortunate and severely deprived women. Despite several supplementary progress interferences, microfinance initiatives are largely rural-focused and designed to elevate destitute women. Microfinance is generally regarded as a powerful instrument for alleviating poverty and promoting self-sufficiency among women by providing financial services without requiring collateral. Women contribute to cutting-edge MFIs, which often reflect important developments in revenue generation, savings behavior, and decision-making within the domestic and public sectors. It was found that microfinance has been associated with improved financial position, greater input in household and municipal outcomes, and better self-esteem and information among women. Notwithstanding the reality of frequent curricular shortages, microfinance is one of the most effective instruments for empowering rural women and encouraging self-employment. It portrays a transformative character by allowing womenfolk to assume limited commercial roles, thus improving their socio-economic situation. Generally, the research reveals a durable positive association between microfinance facilities and various levels of women’s empowerment, emphasizing the importance of microfinance as a means of communal development and gender equality.

Nguyen (2020) examined the impact of CAR on the profitability of Vietnamese commercial banks. It was mainly based on the Basel II outline, which used data from 22 banks from 2010 to 2018. This research used panel data with regression, although the study focused on variables such as net interest margin, non-performing loans, and ownership configuration. It was found that the CAR is completely prejudiced against the ROA, i.e., smaller banks; nevertheless, it has no effective impact on the superior banks. Government ownership and non-performing loans have negatively affected profitability. This research concluded that capital adequacy plays an additional perilous role for minor banks; moreover, supervisory acquiescence alone might not guarantee effectiveness in larger companies.

Shabani et al. (2019) examined the association between capital adequacy and ROA in Kosovo’s banking segment, using secondary data from 2008 to 2017. This study was practical, immovable, and accidental, using special-effects models with the GMM technique. It was found that there was an important positive association between capital adequacy and ROA. It was recommended that well-capitalized banks remain strong and money-spinning. Supplementary bank-specific variables similarly influenced ROA both positively and negatively. This research provides empirical evidence that capital adequacy, which was the main factor in the economic performance of emerging banking markets alike in Kosovo, is a key factor in economic performance.

Datta and Mahmud (2018) assessed the association between capital adequacy and profitability in 29 listed commercial banks in Bangladesh using data from 2007 to 2014. Across 232 samples, the panel regression methods remained useful. This study found that the banks mostly preserved supervisory capital beyond Basel II requirements. The key factors affecting profitability include capital adequacy, operational efficiency, asset size, and loan construction. The study found a positive relationship among CAR, ROA, and ROE, with a focus on the role of strong capital stations in maintaining economic stability.

Taherinia and Baqeri (2018) evaluated the impact of CAR on bank capital in Iranian banks registered on the Tehran Stock Exchange. It was enclosed from 2009 to 2013. After exhausting regression models and numerical tests, the exploration assessed the association between CAR and influences such as bank size, interest rates, and profit instability. It was indicated that the CAR has positive implications for bank reserves, suggesting higher capital cushions to enhance financial stability. The bank size, besides the capability charges, had negative effects. There were development opportunities and profit instability due to positively impacted reserve heights, which was settling the strength of the capital-reserve association.

Almazari and Alamri (2017) studied the impact of capital adequacy on profitability by associating two main Saudi Arabian banks, SAMBA and SABB. Descriptive statistics were used in addition to correlation analysis. The relationships among many capital ratios and profitability indicators, such as ROA and ROE, were studied. The aim at SABB, ROA was completely related to core capital ratios and negatively affected by asset growth and liquidity. The SAMBA company shows a strong positive correlation between ROA and ROE. Although capital adequacy is frequently negatively associated with effectiveness. The consequences propose bank-specific approaches that meaningfully inspire capital profitability, which is self-motivated. Table 1 summarizes the empirical review.

**Table 1.** Summary of empirical review.

Authors and date	Findings
Bei et al. (2025)	There were higher capital adequacy ratios (CAR) maintained by FinTech acceptance, with recoveries on financial resilience and profitability (ROA, ROE, NIM). Though very high CAR levels yield diminishing returns, indicating that a balance is desirable between capital cushions and operational efficacy.
Ikpesu and Oke (2025)	An important negative association was initiated between the capital adequacy ratio (CAR) and bank profitability (ROA) in Nigerian banks. CAR improves the financial stability; nevertheless, it might limit giving capacity and reduce earnings.
Johnson et al. (2025)	It was noted that a higher CAR improves the flexibility and profitability (ROA, NIM) of U.S. banks; nonetheless, a very high CAR can diminish advance and profitability without a balance between stability and growth.
Liza (2025)	It was found that in Bangladeshi banks, capital adequacy negatively influences profitability. Although asset quality and other factors, such as GDP growth, positively affect profitability. There were significant fluctuations in post-Basel improvements.
Thapa et al. (2025)	It was found that the superior and additional profitable Nepalese banks maintain higher CAR, although increased non-performing loans and payments reduce capital adequacy, highlighting the need for a balance between profitability and risk regulation.
Warasto and Janudin (2025)	There was also an examination of the higher CAR, which reflects the financial performance of an Indonesian bank, emphasizing the importance of durable capital defenses for sustainable success.
Humta et al. (2024)	There was also an increase in undesirable effects on bank profitability. Capital adequacy, exchange rates, and interest rates significantly affect profitability. The macroeconomic variables play a significant role in the relationship between capital adequacy and profitability.
Kunjeda (2024)	The capital adequacy ratios of Nepalese banks meet regulatory requirements, but profitability ratios show a declining trend. Capital adequacy has a weak positive correlation with profitability, but no significant effect on profitability according to regression analysis.
Thapa (2024)	Revealed positive effects of total CAR and Tier I capital on profitability measures (ROA, EPS) in Nepalese banks, while Tier II capital and high deposits had negative effects. Non-performing loans reduced profitability.
Chalise (2023)	A negative correlation is observed between capital adequacy ratios (CARs) and profitability measures such as ROA and ROE. Factors such as the Debt-Equity Ratio (D-ER) positively influence profitability, while ratios such as Non-Performing Loans (NPLR) negatively affect it.
Goet (2022)	Shareholders’ equity and Tier 1 capital are positively related to profitability, while Tier 2 capital has no significant effect. The credit-deposit ratio significantly impacts ROA, and both shareholders’ equity and CAR affect ROE.
Mamun et al. (2022)	A negative relationship exists between CAR and ROA, and between CAR and ROE. The credit-deposit ratio (CDR) was positively correlated with ROE. Capital adequacy is significantly related to both ROA and ROE, highlighting its importance in bank recapitalization.
Anggraini and Suputra (2021)	There was a positive relationship between capital adequacy and liquidity, and between profitability and CAR. Where credit risk has a destructive impact on profitability.
Bata et al. (2021)	There was also a significant impact of capital and liquidity on profitability. There were credit subjects that were not prepared properly, focusing on profitability rather than the sensible effect of adequacy.
Jadhav et al. (2021)	The CAR ratio influences profitability, with measurable effects on profitability indicators. Efficient capital management policies are suggested to achieve attractive performance.
Indriani et al. (2020)	There was a tentative negative effect of CAR on ROA. TPF knowingly increases success by refining and advancing events.
Lamichhane (2020)	Microfinance significantly improves women’s financial position, executive power, self-employment opportunities, and overall empowerment.
Nguyen (2020)	The smaller banks’ profit is amplified by the capital adequacy, refining ROA, although superior banks display no significant impact.
Shabani et al. (2019)	There was a positive effect of the CAR on ROA. Additional issues also affect ROA; the consequences vary across different variables.
Datta and Mahmud (2018)	The variables CAR sideways through operational efficiency and loan assembly were positively associated with profitability, representing the position of capital organization.
Taherinia and Baqeri (2018)	Similarly, the CAR was positively correlated with bank reserves. Influences such as growth opportunities and profit instability have positively affected reserves, whereas settled services and bank size have negatively affected them.
Almazari and Alamri (2017)	The SAMBA Bank shows a strong positive relationship between ROA and ROE, whereas the SABB Bank shows relationships that vary with fiscal ratios, such as CAR, in addition to equity capital.

### 3.3 Research gap

These were the gaps in the research on the microfinance sector in Nepal. It was mainly in relation to capital adequacy management, which was significant. Though earlier studies have examined capital adequacy and performance in commercial banks. There was an imperfect study on Nepalese microfinance institutions (NMFIs) around that time. The prevailing fiction primarily revolves around superior financial institutions, creating a fissure of sympathy that has hindered MFIs from meeting their capital adequacy requirements in line with supervisory guidelines.

The research gap in the Nepalese microfinance segment was most evident when considering wholesaler and retailer microfinance institutions (MFIs); this division has been largely overlooked in the present literature. Whereas previous research has focused on capital adequacy in commercial banks or on extensive research on MFIs, there has been a lack of intensive investigation into wholesaler MFIs such as First Microfinance, Sana Kisan Bikas, and RSDC to understand the capital adequacy associated with retailer MFIs such as Chhimek, Nirdhan Utthan, and Forward Microfinance. Wholesaler MFIs mainly lend to smaller institutions, while retailers contract directly with end clients, subsequently adopting different operational models, risk profiles, and capital management approaches. This study examines these gaps by purposively sampling 10 MFIs that represent both groups to assess the effect of capital adequacy agendas, such as the Capital Adequacy Ratio (CAR), on profitability, which differs significantly across institutional types. It was found that the study’s detection suggests an original influence on the microfinance fiction in Nepal, if proportional intuitions from preceding exploration have not been satisfactorily transmitted.

Previous research by Humta et al. (2024) and Mamun et al. (2022) focuses primarily on commercial banks, examining the impact of capital adequacy, profitability, and overall economic performance. Nevertheless, this research does not discuss the exclusive contests and instruments used by microfinance institutions. For instance, research conducted by Chalise (2023) and Kunjeda (2024) examined capital adequacy and profitability among commercial banks in Nepal; similar studies do not extend to microfinance institutions operating under diverse financial constructs and regulatory constraints.

Moreover, Nepal Rastra Bank’s overview of new supervisory contexts, such as the 2015 capital adequacy framework, which comprises the leverage ratio, countercyclical cushion, and capital preservation bumper. It has not been sufficiently discussed in the prevailing literature ( Kunjeda, 2024). The operation of these agendas has yet to be studied with reference to microfinance institutions. There was similarly an absence of studies focusing on the internal instruments and practices that microfinance institutions use to achieve capital adequacy.

This research covers the three microfinance institutions, Nirdhan Utthan Microfinance, Forward Microfinance, and Chhimek Microfinance, that seek to seal this opening. These institutes provide a representative example of many working families and stockholders, similar to the method used in previous studies of commercial banking ( Goet, 2022). The research was applied to statistics after the historical 2014/015 to 2023/024. The concentration on serious financial relations like the core capital ratio (CCR), supplementary capital ratio (SCR), capital adequacy ratio (CAR), cash reserve ratio (CRR), leverage ratio (LR), return on assets (ROA), and return on equity (ROE).

By addressing these gaps, this investigation aims to provide an understanding of the capital adequacy performance of microfinance institutions in Nepal and to evaluate the impact of capital adequacy on their economic performance.

### 3.4 Research hypotheses

Based on the empirical review following hypotheses were formulated.

H _1_: There is a significant impact of capital adequacy ratio on return on assets and return on equity of Nepalese microfinance institutions (NMFIs).

H _2_: There is a significant impact of core capital ratio on return on assets and return on equity of NMFIs.

H _3_: There is a significant impact of the supplementary capital ratio on return on assets and return on equity of NMFIs.

H _4_: There is a significant impact of cash reserve ratio on return on assets and return on equity of NMFIs.

H _5_: There is a significant impact of leverage ratio on return on assets and return on equity of NMFIs.

**Research framework**

**Figure 1.**
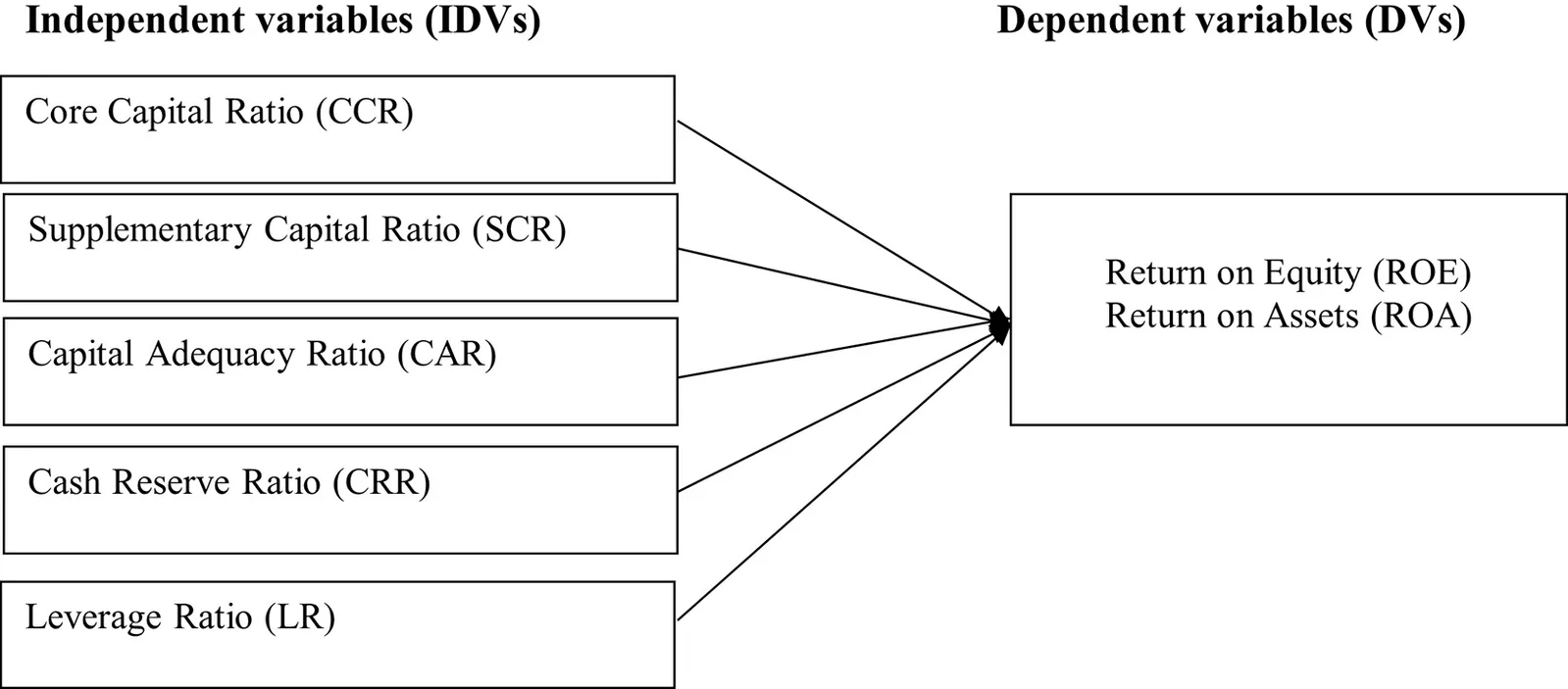
Research framework. *Source*: Kunjeda, 2024.

In the model, the capital adequacy ratio and its impact on profitability are explained by seven explanatory variables and a control. Figure 1 illustrates the conceptual framework depicting the relationship between the capital adequacy ratio and profitability. Figure 1 depicts the research framework for the study.

Figure 1 presents the study outline of the research. This research framework examined the core capital ratio, supplementary capital ratio, capital adequacy ratio, cash reserve ratio, and leverage ratio as independent variables, with return on assets and return on equity as dependent variables.

## 4. Research methodology

### 4.1 Research design

This research uses the descriptive and causal-comparative investigation method. The data were used to evaluate the impact of the capital adequacy ratio on profitability in Nepalese microfinance institutions and to inform future research. A causal-comparative investigation design was applied to assess the association between capital adequacy ratios and profitability in Nepalese microfinance institutions. The study followed a quantitative approach.

### 4.2 Population and sample, and sampling design

In Nepal, there are 57 registered MFIs as of fiscal year 2082/2–10. For this study, 10 microfinance institutions were purposively selected based on their significant presence, influence, and financial diversity. The sampling is structured to capture both wholesaler and retailer microfinance institutions, reflecting different operational scales and business models. Specifically, FMLBSL, SKBLBSL, and RLBSL are categorized as wholesaler microfinance institutions, accounting for 30% of the total sample. These wholesalers primarily provide bulk loans to smaller microfinance institutions or cooperatives rather than directly to individual clients.

The remaining 70% of the illustration involves seven retail microfinance institutions, including CLBSL, NULBSL, FMLBSL, GBLBSL, MMLBSL, NMBLBSL, and NADEPLBSL. Those institutes involve direct end customers, provided they are limited to entities and collections for industrious investment decisions.

This sample method safeguards a representative sample of large-scale, extensive creditors and smaller-scale, retail-oriented microfinance breadwinners, enabling a comprehensive and proportional examination of the capital adequacy ratio (CAR) and its impact on profitability across diverse administrative categories within Nepalese microfinance institutions.

### 4.3 Nature and sources of data

The main source of the data was secondary, comprising the annual reports of the selected microfinance institutions. It was providing the thorough financial statements essential for analyzing capital adequacy and profitability. Furthermore, the research combined data and intuitions, drawing on both published and unpublished sources. Applicable resources such as academic journals, newspapers, and magazines were studied to capture broader industrial tendencies and background empathy. Additionally, official reports and catalogues from the Nepal Stock Exchange (NEPSE), Merolagani, Sharesansar, and the NRB are important sources of consistent financial data and regulatory information.

### 4.4 Data collection instruments and procedure

The data collected from the annual reports of the selected institutions and from reports published by the Nepal Rastra Bank were organized and analyzed in Excel. The use of suitable data collection methods enhanced the credibility and value of the research outcomes.

### 4.5 Data analysis tools and methods

Financial and statistical techniques, such as mean, standard deviation, correlation, and multiple regression, were used to analyze data from the sampled institutions’ annual reports.

### 4.6 Financial tools

In the financial ratio return on assets, return on equity, core capital ratio, supplementary capital ratio, capital adequacy ratio, cash reserve ratio, and leverage ratio of the company were included. This study employs both simple and multiple regression analyses to examine and test the relationship between the dependent and independent variables. Simple regression assesses the relationship between one dependent variable and one independent variable, whereas multiple regression analyzes the relationship involving more than one independent variable.

Regression models serve to evaluate the relationship between a dependent variable and one or more independent variables. The specific regression model used in this study has been analyzed accordingly.

$$\mathrm{ROE}=a+b_{1}\times \mathrm{CCR}+b_{2}\times \mathrm{SCR}+b_{3}\times \mathrm{CAR}+b_{4}\times \mathrm{CRR}+b_{5}\times \mathrm{LR}$$

$$\mathrm{ROA}=a+b_{1}\times \mathrm{CCR}+b_{2}\times \mathrm{SCR}+b_{3}\times \mathrm{CAR}+b_{4}\times \mathrm{CRR}+b_{5}\times \mathrm{LR}$$

ROE = Return on Equity.

ROA = Return on Assets.

CCR = Core Capital Ratio.

SCR = Supplementary Capital Ratio.

CAR = Capital Adequacy Ratio.

CRR = Cash Reserve Ratio.

LR = Leverage Ratio.

## 5. Results and discussion

In this study, the dependent and independent variables were included. Among them, return on assets and return on equity are dependent variables, where core capital ratio, supplementary capital ratio, capital adequacy ratio, cash reserve ratio, and leverage ratio were independent variables.

### 5.1 Descriptive analysis

Table 2 presents descriptive statistics for the variables.

**Table 2.** Descriptive statistics of the variables.

Variables	N	Minimum	Maximum	Mean	Std. deviation
ROA	100	−1.88	4.48	1.6331	1.23999
ROE	100	1.37	38.67	13.4108	8.71439
CCR	100	1.92	34.00	11.5885	4.32022
SCR	100	.41	1.80	1.0893	.33251
CAR	100	2.73	35.00	12.6828	4.37641
CRR	100	.01	3.27	1.1617	.72711
LR	100	−54.66	1153.00	40.8971	159.3828

*Source:* Annual report of selected microfinance.

Table 2 examined 7 selected study variables from sampled institutions, with data collected over 100 years or a sample size of 100. The study variable, return on assets, ranges from −1.88 to 4.48, with a mean of 0.68 and a standard deviation of 1.03, across the sampled institutions for the concerned periods. Similarly, ROA has a mean of 1.6331 and a standard deviation of 1.23999. It indicated that the sample institutions were achieving an overall effective return on their total assets.

Similarly, another variable, return on equity, ranges from 1.37 to 38.67, with an average of 134108 and a standard deviation of 8.71439. The overall capacity of ROE was satisfactory, but some institutions had the lowest equity, while others had the highest. The institutions that have the lowest must expand their operations effectively and take profitable steps. In the same way, the variable core capital ratio showed that the minimum value for sample institutions was 1.92, the maximum was 34.00, the average was 11.5885, and the standard deviation was 4.32022, indicating no high risk to capital.

From the perspective of the supplementary capital ratio, the average ratio was 1.0893, with a standard deviation of 0.33251, indicating no high risk in the sample institutions’ capital structures. There were .41 minimum supplementary capital ratio and a 1.80 maximum supplementary capital range. This data showed relatively consistent servicing of the total capital. The capital adequacy ratio ranges from 2.30 to 35.00 for the sample institutions. The average ratio was 12.6828, with a standard deviation of 4.37641, indicating lower risk on the CAR, which is satisfactory. The study of another variable, the cash reserve ratio, indicated that, overall, they have maintained their reserves, but some individual institutions have not. The minimum CRR was 0.01, with a maximum of 3.27 percent; the average ratio was 1.1617, and the standard deviation was 0.72711. At last, the variable leverage ratio whose minimum range was −54.66 and maximum range was 1153.00, whose average range was 40.8971, and standard deviation was 159.3828. The standard deviation reflected significant inequalities in the debt financing requirements; some sampled institutions were heavily leveraged, while others had minimal or negative leverage.

### 5.2 Correlation analysis

The correlation analysis examined the relationship between the study variables. The variables were measured as positive or negative, effective or not. In the study, the variables were return on assets, return on equity, core capital ratio, supplementary capital ratio, capital adequacy ratio, cash reserve ratio, and leverage ratio, which were examined for their relationships. Table 3 indicates the correlation matrix.

**Table 3.** Correlation matrix.

Variables	ROA	ROE	CCR	SCR	CAR	CRR	LR
ROA	1						
ROE	.907 ^**^	1					
	(.000)						
CCR	.147	.117	1				
	(.144)	(.246)					
SCR	.355 ^**^	.414 ^**^	.132	1			
	(.000)	(.000)	(.189)				
CAR	.168	.143	.697 ^**^	.207 ^*^	1		
	(.094)	(.156)	(.000)	(.039)			
CRR	.472 ^**^	.447 ^**^	.076	.409 ^**^	.103	1	
	(.000)	(.000)	(.455)	(.000)	(.306)		
LR	−.282 ^**^	−.140	−.064	−.070	−.067	−.282 ^**^	1
	(.004)	(.166)	(.526)	(.488)	(.506)	(.004)	

*Source:* Annual report of selected microfinance.

** Correlation is significant at the 0.01 level (2-tailed).

* Correlation is significant at the 0.05 level (2-tailed).

The correlation matrix presented in Table 3 shows that the strength and direction of the relationships between profitability indicators, specifically return on assets (ROA) and return on equity (ROE), alongside the explanatory variables. The findings indicate that ROA and ROE are significantly and positively correlated (r = 0.907, p < 0.01), which confirms that both profitability measures tend to move in tandem. This observation suggests that institutions that excel in asset utilization are also likely to deliver substantial returns to equity holders.

They were examining other variables, the core capital ratio (CCR), which shows a weak, statistically insignificant positive relationship with ROA (r = 0.147, p > 0.05) and ROE (r = 0.117, p > 0.05). This study also explained that core capital does not exert a uniform influence on profitability across the appraised institutions. In a comparable manner, the supplementary capital ratio (SCR) exhibits a reasonable positive correlation with ROA (r = 0.355, p < 0.01) and ROE (r = 0.414, p < 0.01), indicating that supplementary capital contributes to attractive profitability through a statistically significant relationship.

The capital adequacy ratio (CAR) exhibits a weak, statistically insignificant positive association with ROA (r = 0.168, p > 0.05), as well as with ROE (r = 0.143, p > 0.05). However, CAR is strongly positively correlated with CCR (r = 0.697, p < 0.01), suggesting that capital adequacy is primarily determined by core capital. Additionally, the cash reserve ratio (CRR) exhibits a significant positive association with ROA (r = 0.472, p < 0.01) and with ROE (r = 0.447, p < 0.01), indicating that institutions that maintain higher reserves are likely to attain higher profitability. Furthermore, CRR is positively and significantly related to SCR (r = 0.409, p < 0.01), underscoring the importance of the connection between the standby organization and SCR, beyond supplementary capital.

Finally, the leverage ratio (LR) shows a negative association with both profitability events. The overtone is significant by ROA (r = −0.282, p < 0.01), nonetheless not significant through ROE (r = −0.140, p > 0.05). This study proposes that increased leverage reduces returns on assets and equity to a lesser degree and highlights the possible risks associated with extreme debt requirements. Furthermore, LR is significantly and negatively associated with CRR (r = −0.282, p < 0.01), indicating that institutes with greater influence naturally preserve lower reserve levels.

It was determined that the effectiveness of Nepalese microfinance institutions is clearly linked to supplementary capital and cash reserves, whereas extreme leverage adversely affects returns. Although core capital and capital adequacy are critical in a supervisory position, they do not explain the significant direct associations with profitability. The durable association between ROA and ROE supports the consistency of these profitability metrics in official presentations.

### 5.3 Regression analysis

The next segment focuses on regression analysis, which investigates the effect of the capital adequacy ratio on the profitability of Nepalese microfinance institutions. The dependent variables for this examination are return on assets (ROA) and return on equity (ROE); however, the independent variables comprise the core capital ratio (CCR), supplementary capital ratio (SCR), capital adequacy ratio (CAR), cash reserve ratio (CRR), and leverage ratio (LR). The multiple regression replicas were used to assess the extent to which these independent variables explain the disparities in the dependent variables. The model summary of ROA is presented in Table 4.

**Table 4.** Model summary of ROA.

Model	R	R square	Adjusted R-square	Std. error of the estimate
1	.561	.315	.279	1.05315

a. Predictors: (Constant), LR, CCR, CRR, SCR, CAR.

*Source:* Annual report of selected microfinance.

Table 4 summarizes the model applied in the reversion examination, with return on assets (ROA) as the dependent variable. The independent variables include the leverage ratio (LR), core capital ratio (CCR), cash reserve ratio (CRR), supplementary capital ratio (SCR), and capital adequacy ratio (CAR).

The results indicate that the model exhibits a correlation coefficient (R) of 0.561, indicating a reasonable positive correlation between the independent variables and ROA. The coefficient of determination (R-squared) is 0.315, indicating that almost 31.5% of the variation in ROA is explained by the nominated independent variables. Following a modification to the number of interpreters, the Adjusted R^2 decreases only slightly to 0.279, which remains a considerable measure of the model’s descriptive ability. The standard error of the estimate is 1.053, indicating that the forecast ROA values deviate, on average, by roughly 1 percentage point from the empirical standards. It was evident that the nominated financial ratios provide a modest clarification of the profitability of microfinance institutions relative to ROA. Though the model does not fully capture the influence of capital structure, investments, and leverage on returns on assets, it demonstrates that these factors collectively exert a significant influence on them. Table 5 presents the ANOVA of ROA.

**Table 5.** ANOVA of ROA.

Model		Sum of squares	df	Mean square	F	Sig.
1	Regression	47.962	5	9.592	8.649	.001
	Residual	104.257	94	1.109		
	Total	152.220	99			

a. Dependent Variable: ROA;

b. Predictors: (Constant), LR, CCR, CRR, SCR, CAR.

*Source:* Annual report of selected microfinance.

Table 5 presents the findings of the analysis of variance (ANOVA) for the regression model. The return on assets (ROA) was examined as the dependent variable, and the predictors included the leverage ratio (LR), core capital ratio (CCR), cash reserve ratio (CRR), supplementary capital ratio (SCR), and capital adequacy ratio (CAR). The regression sum of squares amounts to 47.962 through 5 degrees of freedom, where the residual sum of squares totals 104.257 with 94 degrees of freedom. The calculated mean square for regression is 9.592, compared with 1.109 for the residuals.

The F-statistic is considered at 8.649, through a conforming significance level of 0.001. They were significantly lower than the 5% threshold. It was suggested that the overall regression model, which is statistically significant, represents the grouping of independent variables and provides a considerable explanation for the differences observed in ROA.

The ANOVA results indicate that the descriptive variables, including capital ratios, reserve ratios, and leverage, together exercise a significant effect on the return on assets of microfinance institutions. The model demonstrates a good fit, and the predictors effectively explain profitability. The coefficients of ROA are presented in Table 6.

**Table 6.** Coefficients of ROA.

Model		Unstandardized coefficients		Standardized coefficients	t	Sig.
		B	Std. error	Beta		
1	(Constant)	4.501	.973		4.624	.000
	CCR	2.622	1.360	9.134	1.928	.057
	SCR	3.348	1.418	.898	2.361	.020
	CAR	−2.597	1.360	−9.167	−1.910	.059
	CRR	.541	.167	.317	3.237	.002
	LR	−.001	.001	−.161	−1.800	.075

a. Dependent Variable: ROA.

*Source:* Annual report of selected microfinance.

Table 6 presents the regression coefficients aimed at the aspects influencing return on assets (ROA). The continuous term is 4.501 and is statistically significant (p = 0.000), indicating that all independent variables are set to zero; the starting point ROA value remains positive. Among the analysts, the core capital ratio (CCR) has a positive coefficient of 2.622, indicating that an increase in core capital improves ROA. However, its effect is only marginally significant (p = 0.057), indicating that although the relationship is positive, the effect does not meet the conventional 5% significance threshold.

The supplementary capital ratio (SCR) discloses a positive and significant coefficient of 3.348 (p = 0.020), as evidenced by a standardized beta of 0.898. This specifies that supplementary capital plays a considerable role in attracting returns on assets through its statistically significant and practically applicable impact. Conversely, the CAR yields a negative coefficient of −2.597, with a p-value of 0.059. This suggests that higher overall capital adequacy is associated with lower asset returns, possibly due to the opportunity cost of holding excess capital. Though the similarity to CCR is a weakly significant consequence. The cash reserve ratio (CRR) expresses a positive and significant influence on ROA (B = 0.541, p = 0.002). Through a standardized beta of 0.317, which specifies that amplified cash reserves are related to improved profitability, underscoring the importance of liquidity management in attractive asset returns.

Finally, the leverage ratio (LR) exhibits a negative coefficient (B = −0.001), with a p-value of 0.075, indicating that increased leverage is associated with a decrease in ROA, though this effect is only statistically significant at the 10% level. This remark suggests that overdependence on debt-fueled bankrolling might reduce profitability. The coefficient analysis shows that supplementary capital and cash reserves meaningfully contribute to ROA; however, high capital adequacy and leverage negatively affect profitability. Core capital displays a positive yet feeble influence. These results highlight the need for evenness that microfinance institutions are obligated to achieve in strengthening their capital structures, managing reserves, and navigating the challenges posed by extreme debt to improve asset returns. The model summary of ROE is presented in Table 7.

**Table 7.** Model summary of ROE.

Model	R	R square	Adjusted R-square	Std. error of the estimate
1	.548	.301	.264	7.47856

a. Predictors: (Constant), LR, CCR, CRR, SCR, CAR.

*Source:* Annual report of selected microfinance.

Table 7 presents the regression model for return on equity (ROE) using LR, CCR, CRR, and SCR as predictors, with CAR as the dependent variable. The correlation coefficient (R) is 0.548, indicating a modest positive relationship between the set of independent variables and ROE.

The coefficient of determination (R ^2^) is 0.301, indicating that almost 30.1% of the variation in ROE is explained by these financial ratios. Subsequently, adjusting for the number of analysts, the Adjusted R ^2^ is somewhat lower at 0.264, indicating that the model explains only a small portion of the variation in equity returns. The standard error of the estimate is 7.479, indicating that the predicted ROE values deviate from the experimental values by about 7.48 units on average, suggesting moderate accuracy in the model’s predictions.

The consequences specify that capital structure, reserves, supplementary capital, and influence, together, have a reasonable descriptive influence on the profitability of microfinance institutions in the ROE relationship. Though the model does not encompass all factors affecting ROE, it provides a meaningful basis for understanding the financial relations related to shareholder returns. ANOVA of ROE is presented in Table 8.

**Table 8.** ANOVA of ROE.

Model		Sum of squares	df	Mean square	F	Sig.
1	Regression	2260.801	5	452.160	8.085	.001
	Residual	5257.316	94	55.929		
	Total	7518.116	99			

a. Dependent Variable: ROE

b. Predictors: (Constant), LR, CCR, CRR, SCR, CAR.

*Source:* Annual report of selected microfinance.

Table 8 explains the outcomes of the analysis of variance (ANOVA) for the regression model. The variable return on equity (ROE) was the dependent variable; besides LR, CCR, CRR, SCR, and CAR were the independent variables. The reversion sum of squares is 2260.801 through 5 degrees of freedom, although the residual sum of squares is 5257.316 through 94 degrees of freedom. The consistent mean squares are 452.160 for regression and 55.929 for residual.

It was examined that the F-statistic is 8.085 at an implication level of 0.001, which is below the 5% threshold. This specifies that the complete regression model is statistically significant, in the sense that the set of independent variables together provides an informative explanation of the differences in ROE. The ANOVA results indicate that the mixture of leverage, capital ratios, cash reserves, and supplementary capital significantly influences stockholder returns in microfinance institutions. It was found that these financial factors, measured and compiled, are important drivers of ROE and support further inspection of individual charities for each analyst. Table 9 shows the ROE coefficients.

**Table 9.** Coefficients of ROE.

Model		Unstandardized coefficients		Standardized coefficients	t	Sig.
		B	Std. Error	Beta		
1	(Constant)	42.110	8.682		4.850	.001
	CCR	20.491	9.655	10.159	2.122	.036
	SCR	27.726	10.071	1.058	2.753	.007
	CAR	−20.382	9.655	−10.236	−2.111	.037
	CRR	3.601	1.187	.300	3.033	.003
	LR	−.001	.005	−.018	−.199	.842

a. Dependent Variable: ROE;

*Source:* Annual report of selected microfinance.

Table 9 examines the regression coefficients for the causes of return on equity (ROE). The constant tenure is 42.110, which is statistically significant (p = 0.001), indicating that, after all independent variables are zero, the zero ROE remains positive. The core capital ratio (CCR) has a positive coefficient of 20.491 (p = 0.036), indicating that developed core capital is associated with higher ROE. This relationship is statistically significant at the 5% level, indicating that core capital contributes significantly to shareholder revenues.

The supplementary capital ratio (SCR) showed a positive and significant effect (B = 27.726, p = 0.007), with a consistent beta slightly above 1 (β = 1.058). This implies that supplementary capital plays a significant role in attracting ROE and has a strong and statistically significant influence on both. In contrast, the capital adequacy ratio (CAR) shows a negative coefficient of −20.382 (p = 0.037), indicating that higher capital adequacy is associated with lower ROE. This might reproduce the occasion cost of holding excess capital, such as funds tied up in capital cushions that are not actively organized for revenue-generating events.

The cash reserve ratio (CRR) has a positive and significant effect on ROE (B = 3.601, p = 0.003; β = 0.300), indicating that developed assets provision profitability, provides financial stability, and the ability to meet responsibilities without affecting working efficacy.

Lastly, the leverage ratio (LR) has a negative coefficient (B = −0.001), which is statistically insignificant (p = 0.842). There was a representative in this sample for whom leverage had no expressive impact on shareholder returns. The coefficient analysis for ROE shows that core and supplementary capital together positively influence shareholder returns, while higher total capital adequacy has a diminishing effect. Cash reserves likewise sustain profitability, while leverage does not significantly affect ROE in these institutions. These findings highlight the importance of stable capital management, alongside reserve policies, for attractive equity returns, while deterring the inconsistency of extreme capital cushions that might reduce profitability.

## 6. Discussion, conclusion, and policy implications

### 6.1 Discussion

This research explored the effect of core capital (CCR), supplementary capital (SCR), capital adequacy (CAR), cash reserve ratio (CRR), and leverage ratio (LR) on the profitability of Nepalese microfinance institutions. Which is unhurried, measured by return on assets (ROA) rather than return on equity (ROE)? The results show that supplementary capital and cash reserves significantly improve ROA and ROE together, indicating that institutions with greater additional capital and higher cash reserves tend to achieve higher profitability. Core capital demonstrates a positive yet slightly significant effect, suggesting that although it prepares underwriting for profitability, its influence is not as durable. Equally, capital adequacy shows a negative correlation with profitability, and leverage exhibits a negative, albeit largely insignificant, effect, particularly on ROE. Together, these findings underscore the urgent need for a comprehensive method for capital structure, liquidity management, and debt operations to restore financial performance.

The helpful inspiration of supplementary capital for profitability investigation in this study aligns with previous studies indicating that supplementary capital is central to ornamental financial performance ( Thapa, 2024; Johnson et al., 2025; Warasto & Janudin, 2025). Similarly, the auspicious effect of cash assets is consistent across studies that highlight the implications of liquidity for profitability ( Anggraini & Suputra, 2021; Bata et al., 2021; Humta et al., 2024). The slightly positive influence of core capital similarly aligns with preceding studies that propose a strong equity improperly recovers returns ( Thapa, 2024; Goet, 2022; Jadhav et al., 2021).

The converse relationship between capital adequacy and profitability recognized in this study is validated by frequent earlier findings. This investigation indicates that although a high level of capital adequacy strengthens financial stability, it may constrain lending capacity and operational efficiency, thereby weakening profitability ( Ikpesu & Oke, 2025; Liza, 2025; Chalise, 2023; Mamun et al., 2022). It implies that unnecessary capital investments could experience opportunity costs, as funds allocated to capital are not accessible for income-generating undertakings. Similarly, the incomplete effect of influence on profitability aligns with previous studies that recommend that augmented debt does not inherently improve returns and may increase financial risk ( Mamun et al., 2022; Indriani et al., 2020; Jadhav et al., 2021).

However, some differences arise when comparing these answers with those from other studies. Convinced studies designate a commonly positive association between capital adequacy and profitability, suggesting that a higher Capital Adequacy Ratio (CAR) augments returns ( Bei et al., 2025; Johnson et al., 2025; Warasto & Janudin, 2025; Datta & Mahmud, 2018). This deviation might be credited to related alterations in official size, supervisory agendas, and operational priorities. In the Nepalese microfinance sector, unreasonably high capital adequacy might constrain flexibility, thereby negatively impacting returns. Correspondingly, some prior studies find that leverage and Tier II capital exert significant positive influences on profitability ( Shabani et al., 2019; Goet, 2022). Although this study discovers that influence does not have a statistically significant effect on ROE, which represents the influence of debt to profitability, it is limited to smaller monetary institutions.

The consequences are supported by other studies that highlight the importance of achieving an equilibrium between capital management and operational efficiency. For example, exploration indicates that organizations that maintain appropriate levels of capital, assets, and supplementary reserves can attain superior profitability while efficiently managing risk ( Thapa et al., 2025; Bata et al., 2021; Taherinia & Baqeri, 2018; Jadhav et al., 2021). This evenness empowers institutions to endure monetarily vigorous without unreasonably investing in capital that does not yield direct returns.

Furthermore, the beneficial effects of cash reserves identified in this investigation corroborate findings that maintaining adequate liquidity enables institutions to meet short-term obligations, mitigate operational disruptions, and preserve profitability under adverse economic conditions ( Humta et al., 2024; Anggraini & Suputra, 2021).

Besides, the results of this study underscore the multifaceted association between capital adequacy and profitability, which varies by capital category. While supplementary and core capital tend to augment returns, the complete capital adequacy ratio (CAR) could exert a negative influence. This aligns with research indicating that Tier I capital and shareholders’ equity positively affect profitability; however, Tier II capital or excessive total capital can reduce returns ( Thapa, 2024; Goet, 2022; Mamun et al., 2022). This highlights the necessity for institutions to precisely match the structure and amount of capital to improve performance.

The research stresses that the profitability of microfinance institutions is not only determined by the sum of capital and investments, but also through the strategic balance between financial stability and operational efficiency. Added capital and cash reserves are crucial influences on ROA and ROE, although high levels of capital adequacy also necessitate cautious management to avoid diminishing returns.

The results align closely with previous studies ( Thapa, 2024; Ikpesu & Oke, 2025; Chalise, 2023; Warasto & Janudin, 2025; Mamun et al., 2022), with minor differences that underscore the implications of context-specific approaches for enhancing economic performance. To recover profitability, microfinance institutions would arrange the establishment of additional principal and the conservation of adequate cash reserves, whereas the rudiments positively affect both ROA and ROE. Core capital requirements must be met carefully to ensure stability without limiting operational flexibility. Though capital adequacy is critical for financial security, excessively high levels can reduce profitability; consequently, institutions must balance capital cushions with lending and investment activities. Leverage must be used cautiously, as excessive reliance on debt can intensify risk without offering improved returns. Fundamentally, an unhurried approach to capital management, liquidity planning, and debt operations helps institutions achieve sustainable and efficient financial performance.

### 6.2 Conclusion

The study concluded that the impact of capital structure, assets, and leverage on the profitability of Nepalese microfinance institutions is mediated by core capital, supplementary capital, capital adequacy, the cash reserve ratio, and the leverage ratio. Profitability ratios included return on assets and return on equity. It was found that the supplementary capital and cash reserves have a significant positive impact on both ROA and ROE. Institutions that maintain higher supplementary capital and adequate liquidity are better positioned to generate consistent returns, underscoring the importance of effective capital and reserve management in relation to economic enactment.

The core capital illustrated that the positive relations have a slightly significant effect, suggesting that although it is indispensable to maintain stability and control, its direct influence on effectiveness is adequate. The capital adequacy ratio is negatively associated with profitability. It was determined that unreasonably high elevation levels might limit active suppleness and reduce the ability to develop returns. The leverage ratio expresses a negative effect on ROA, besides a principally inconsequential impact on ROE. There was an inference that over-reliance on debt backing does not unavoidably recover profitability, but it might increase monetary risk.

This research focuses on the position of preserving a well-adjusted economic plan. Microfinance institutions’ obligation is to improve their combination of additional and core capital, confirm sufficient liquidity, and practice leverage carefully. The positive benefits of supplementary capital and cash reserves advise that tactical distribution of internal reserves and liquidity management can efficiently improve profitability. There was a negative consequence of capital adequacy, and the imperfect impact of leverage reveals that excessively conformist or destructive monetary strategies can weaken returns.

The MFIs may emphasize that it was arranged through the consolidation of supplementary capital, thereby controlling reserves efficiently to recover profitability. The core capital ought to remain at levels that meet supervisory requirements, without limiting operational capacity. Capital adequacy must be prudently maintained to avoid unnecessary cushions that reduce efficiency, and leverage must be carefully managed to sustain growth without signaling excessive risk. By adopting this composed approach, organizations can achieve sustainable effectiveness while maintaining financial stability.

Generally, this study finds that the profitability of NMFIs is primarily determined by the actual deployment of supplementary capital and liquidity. A well-calibrated equilibrium among capital, reserves, and responsibility is indispensable to safeguarding financial flexibility, operational efficiency, and sustainable development. The results indicate that there was strong leadership by recognized executives, in collaboration with policymakers, to implement monetary strategies that improve performance, increase revenues, and preserve stability in the microfinance sector.

### 6.3 Implications

The study were emphasis on core capital ratio (CCR) and leverage ratio (LR) after scheming the principles for microfinance institutions (MFIs). There were encouraging policies that reinforced MFIs’ capital improvement, although with extreme supervisory leverage. There were also evaluations of the prevailing capital adequacy guidelines to confirm they meet the requirements of NMFIs. There was trust in MFIs from side to side, as well as durable capital situations that can mitigate risks amid financial uncertainty. The study was not free from its limitations. Only secondary data was collected. For future researchers, it is recommended to build upon these findings by employing a mixed-methods approach. This study will be useful for regulators, microfinance institutions, investors, scholars, academicians, and economist to abilities policy formulation.

## Ethical approval

Ethical approval is not required because the data are secondary.

## Data Availability

Repository name: Impact of capital adequacy on the profitability of microfinance institutions in Nepal. https://doi.org/10.5281/zenodo.18860868 ( Lamichhane, et al., 2026). The project contains the following underlying data:
• Data used for the research.docx (Data used for the research - Appendix: Years/Variables, ROA, ROE, CCR, SCR, CAR, CRR, LR, Company) There is no extended data. Data are available under the terms of the Creative Commons Attribution 4.0 International license “No rights reserved” data waiver (CC-BY 4.0).
