# Capital Adequacy as a Driver of Profitability: Panel Evidence from Nepalese Wholesale and Retail Microfinance Institutions

**DOI:** 10.12688/f1000research.178815.3

**Published:** 2026-09-29

**Authors:** Basu Dev Lamichhane, Satyanarayan Choudhary, Bharat Ram Dhungana, Manika Shrestha

**Affiliations:** 1 Saraswati Multiple Campus, Tribhuvan University, Kathmandu, Nepal; 2 School of Business, Pokhara University, Pokhara, Nepal; 3 Freelance Researcher, Kathmandu, Nepal

**Keywords:** Capital adequacy, MFIs, profitability, regulatory institution, sustainability

## Abstract

**Background:**

Capital adequacy is essential for the sustainable operation of microfinance institutions (MFIs). MFIs can expand their outreach to low-income and marginalized people if the institutions are sustainable.

**Methods:**

This study is based on secondary data collected from the annual reports of the selected microfinance institutions. A descriptive and causal comparative research design was used. The study sampled 10 microfinance institutions from 57 MFIs, including wholesale and retail. The independent variables were capital adequacy ratio, leverage ratio, size, growth, inflation, and type. The dependent variables were return on assets (ROA) and return on equity (ROE). The purposive sampling method was used.

**Results:**

The panel regression model observed that the regression coefficients for SIZE and GDP growth in the fixed-effect panel regression model, and CAR, LR, SIZE, GDP growth, and TYPE are statistically significant for ROA. This study did not find a statistically significant effect on ROE. Profitability of microfinance institutions is not only determined by the sum of capital and investments but also through the strategic balance between financial stability and operational efficiency. High levels of capital adequacy necessitate restrained management to avoid diminishing returns. The regression coefficient for TYPE is statistically significant; the profitability of Retail MFIs and Wholesale MFIs is different.

**Conclusion:**

Capital structure, asset size, and leverage are determinants of the profitability of microfinance institutions. One remarkable result is that the type of microfinance institutions is statistically significant; the profitability of retail MFIs and wholesale MFIs is different. Wholesale MFIs are more capitalized but less profitable than retail MFIs. This implies that capital position does not necessarily translate into higher profitability. Retail business models in terms of financial performance may not necessarily work in wholesale business models. Capital adequacy is a significant determinant of the type of MFI profitability in Nepal. Policymakers should not apply a one-size-fits-all regulation but develop diverse supervisory and capital adequacy frameworks for wholesale and retail MFIs. This study is particularly useful for regulators and microfinance institutions for policy formulation.

## 1. Introduction

MFIs offer financial services to the unbanked population and play a significant role in socio-economic transformation of low-income people. Expanding financial services, livelihood improvement, and poverty reduction through productive investment and collateral-free lending are key attributes of microfinance ( Dhungana et al., 2023). Sustainability of MFIs largely depends on prudential regulations and capital adequacy that ensure MFIs’ ability to withstand both expected and unexpected losses ( Anggraini & Suputra, 2021). Preserving strong capital adequacy originates from supervisory and capital structure perspective that enables sustained operation of MFIs’ lending ( Abate et al., 2014; Lamichhane, 2020).

CAR confirms the financial stability of MFIs, especially in fostering financial prudence. Profitability, loan portfolio quality, and institution size are important elements of CAR among MFIs ( Bata et al., 2021). The profitability of MFIs strengthens capital growth through expanded capital structures ( Bogan, 2012). Adequate capital levels are shaped by institutional alignment, profitability, supervisory requirements, and operational effectiveness ( Bei et al., 2025).

Capital adequacy supports economic stability in the banking and microfinance sectors. Financial institutions must maintain a minimum level of capital, proportional to their risk-weighted assets, to protect shareholders and preserve complete certainty ( Humta et al., 2024). The CAR is supervisory tool used to assess microfinance institution’s ability to maintain healthy status ( Nguyen, 2020). CAR consists of two main components: Tier 1 capital and Tier 2 capital, contributing to safeguarding against insolvency conditions ( Shabani et al., 2019).

The need to preserve adequate capital is emphasized in frequent supervisory guidance, including the Basel Accords, international standards for economic sector regulation ( Benink & Wihlborg, 2002). Regulatory institutions adopt capital adequacy norms tailored for MFIs to improve financial inclusion while reducing total risk ( Goet, 2022). CAR is indicator of a financial institution’s stability and flexibility, measuring its capital adequacy relative to its risk-weighted assets ( Indriani et al., 2020).

Microfinance institutions exhibit high CAR levels, thereby supporting the sequence between capital adequacy and stability. There are positive associations between profitability and CAR. Liquidity and credit quality matter considerably in determining profitability ( Bata et al., 2021).

It is essential to assess whether profitability underwrites the development of CAR through firming internal capital assets, or if capital necessities themselves overwhelm profitability due to preventive financial illiquidity ( Chalise, 2023). Limited leverage due to high capital requirements can constrain MFIs’ ability to offer competitive loan products and reduce their focus on high-return lending, further affecting profitability ( Nguyen, 2020). Understanding this dynamic is crucial for aligning financial regulation with the growth and durability of MFIs in Nepal.

Given the importance of capital adequacy in microfinance institutions, it has been necessary to study various aspects, the impact, and the status of the capital adequacy norms and standards in the context of Nepal. Various questions arise about the subjects such as (i). What is the current status of the capital adequacy ratio, leverage ratio, size, GDP growth, inflation, and type on profitability of Nepalese MFIs? (ii). What is the relationship between capital adequacy ratio, leverage ratio, growth, inflation, and type on profitability of Nepalese MFIs? (iii). What is the impact of the variables, capital adequacy ratio, leverage ratio, growth, inflation, and type on the profitability of Nepalese MFIs? The aim of this study is to evaluate the impact of capital adequacy ratios on the profitability of microfinance institutions in Nepal.

## 2. Literature review

### 2.1 Theoretical review

A theoretical review summarizes the main concepts associated with capital adequacy and profitability of microfinance institutions. Modern portfolio theory (MPT) was established by Markowitz in 1952. It is found on the impression that a stockholder can conceive an “effective” selection that exploits probable returns aimed at an assumed equal level of risk. This model is primarily applicable to MFIs that emphasize expanding economic assets to mitigate risk. Intended for MFIs, preserving a CAR remains important for managing risks. It includes credit, liquidity, and operational risks that affect operational performance. MPT advises that MFIs can expand their credit selections and secure adequate principal to cushion against probable risks ( Parvin et al., 2020). This theory guides MFIs to maintain CAR which is essential to long-term stability and dependable effectiveness.

The risk-based capital adequacy is established by the Basel Committee on Banking Supervision in 1999. It highlights the economic institutions to preserve capital in balance with the risks related to credit, market, and operational ( Lastra, 2004). MFIs analyze the essential capital created on the institution’s risk-weighted assets to assist in determining the minimum capital required to absorb potential losses. The supervisory system safeguards against monetary shocks, prevents liquidation, and utilizes investors’ funds in the productive sector ( Flannery, 1998). Sustaining CAR, MFIs meet supervisory norms while ensuring stability among investors.

Agency theory was advanced by Michael Jensen and William Meckling in 1976. It examines the relationship between principals (owners) and agents (managers). This theory advises that executives may remain incentivized to take extreme risks or engage in activities that benefit them individually. A resilient CAR can help to maintain adequate equity capital to attract potential borrowers, thereby reducing the need for risky liability support. By preserving an adequate CAR, directors are less likely to incur extreme risks ( Collier & Skees, 2012). This reduces the likelihood of conflict among executives and proprietors as joint gatherings are incentivized to preserve the institution’s financial health. A strong CAR serves as a catalyst in bringing to the forefront the benefits of managers and proprietors, encouraging long-term effectiveness. It fosters collaborative working efficiency and sustainability of organization.

### 2.2 Empirical review

Banks would preserve adequate capital cushions through leveraging technology-driven proficiency to improve performance in developing monitoring environments ( Bei et al., 2025). Ikpesu and Oke (2025) found that CAR remains important for confirming financial stability and protecting banks against credit risks. CAR improves banks’ flexibility to economic shocks and positively affects profitability metrics like return on assets (ROA) and NIM ( Johnson et al., 2025). Banks’ essential balance-monitoring capital requirements are met through planned capital management, thereby improving both financial stability and effectiveness. Company size, GDP growth rate, asset quality, and net interest margin entirely affect profitability, although capital adequacy and inflation rate adversely affect it. Preserving capital adequacy is vital for risk management; excessive regulatory capital requirements may diminish profitability ( Liza, 2025).

Thapa et al. (2025) found that banks with higher profitability tend to maintain higher capital adequacy ratios. Conversely, increased non-performing loans and dividend payouts significantly impeded capital adequacy ratios. Warasto and Janudin (2025) revealed that a complex CAR significantly improves the bank’s financial presentation, underscoring the importance of maintaining robust capital safeguards to ensure stability and profitability. Operational capital management is critical for banks to achieve sustainable financial outcomes. Humta et al. (2024) indicated that capital adequacy, exchange rates, and interest rates have a positive influence on profitability, but inflation has an important, undesirable effect.

CAR and liquidity have positive and significant impacts on profitability. Similarly, the credit risk ratio has a negative effect ( Anggraini & Suputra, 2021). Chalise (2023) observed an inverse correlation between CAR and profitability (ROA and ROE). Mamun et al. (2022) revealed that CAR negatively correlated with both ROA and ROE. It signified that high capital, beyond operating costs, might diminish profitability. Nguyen (2020) found that the CAR is completely insignificant against the ROA, i.e., smaller banks; nevertheless, it has no effective impact on superior banks. Government ownership and non-performing loans negatively affect profitability.

Shabani et al. (2019) observed that there was an important positive association between capital adequacy and ROA. Capital adequacy is a key factor of economic performance in financial institutions. The key factors affecting profitability include capital adequacy, operational efficiency, asset size, and loan construction. The study found a positive relationship among CAR, ROA, and ROE, with a focus on the role of strong capital positions in maintaining economic stability ( Datta & Mahmud, 2018). Taherinia and Baqeri (2018) pointed out that CAR has positive implications for bank reserves, suggesting higher capital cushions to enhance financial stability. The bank size, besides the capability charges, had negative effects. There were development opportunities and profit instability due to positively impacted reserve heights, which was settling the strength of the capital-reserve association.

Earlier studies have examined capital adequacy and performance in commercial banks, but limited studies exist from a microfinance perspective in Nepal. Nepalese microfinance segment was most evident when considering wholesaler and retailer microfinance institutions. This division has been largely overlooked in the present literature. Wholesaler MFIs mainly lend to retail MFIs while retailers contract directly with end clients, subsequently adopting different operational models, risk profiles, and capital management approaches.

Based on the empirical *review, the following* hypotheses were formulated.

H _1_: There is a significant positive impact of capital adequacy ratio on profitability of NMFIs.

H _2_: There is a significant positive impact of leverage ratio on profitability of NMFIs.

H _3_: There is a significant positive impact of institutional size on profitability of NMFIs.

H _4_: There is a significant positive impact of GPD on profitability of NMFIs.

H _5_: There is a significant positive impact of inflation on profitability of NMFIs.

H _6_: There is a significant positive impact of type of MFIs on profitability of NMFIs.

H _7_: There is a significant difference in profitability between wholesale and retail MFIs.

In the model, the capital adequacy ratio and its impact on profitability are explained by six explanatory and a control variable. Figure 1 illustrates the conceptual framework depicting the relationship between the capital adequacy ratio and profitability.

**Figure 1.**
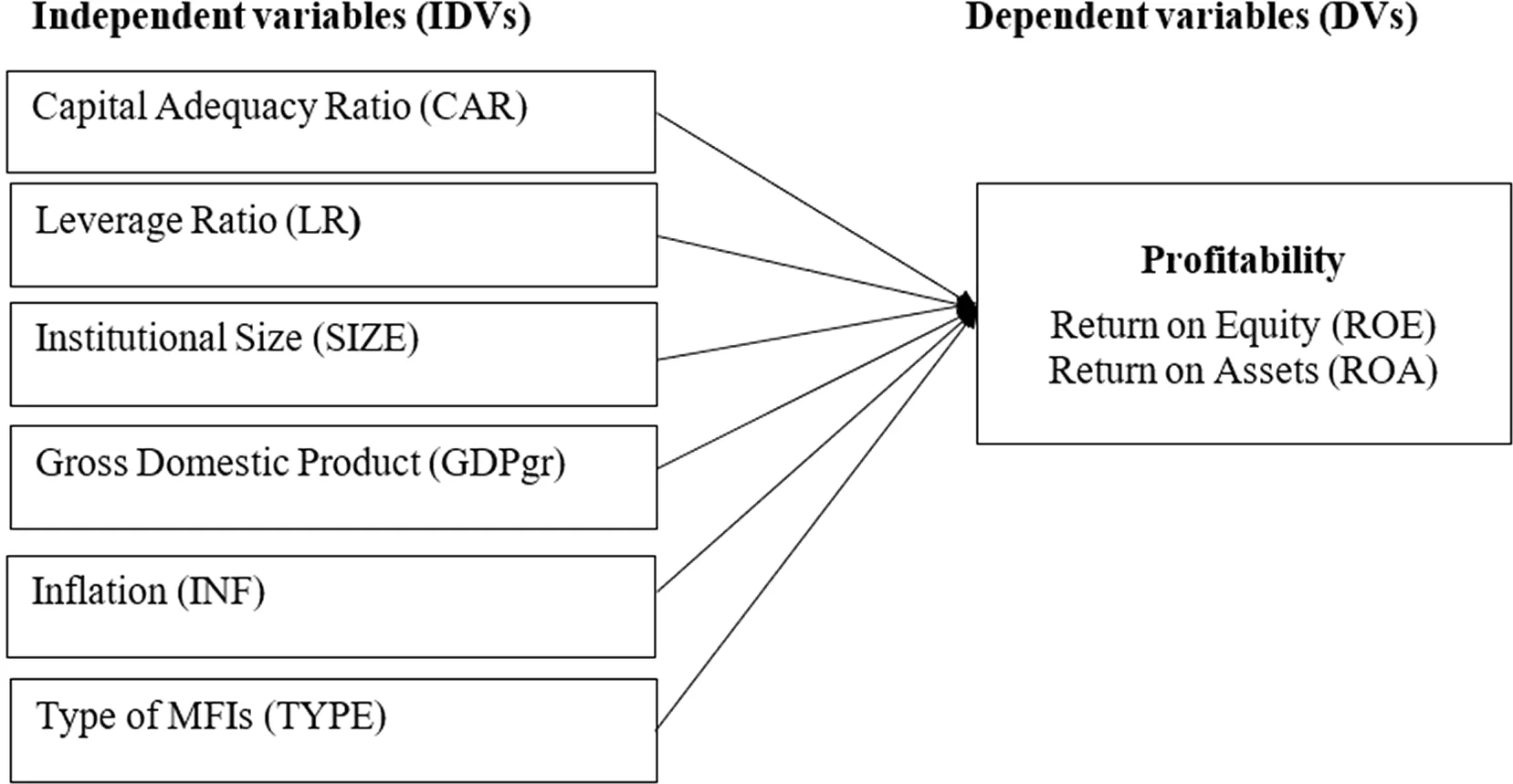
Research framework. Source: Kunjeda, 2024.

Figure 1 presents the study outline. This research framework examined the capital adequacy ratio, leverage ratio, size, GDP growth, inflation, and type of MFIs as independent variables, with return on assets and return on equity as dependent variables.

## 3. Research methodology

### 3.1 Research design

This research uses the descriptive and causal-comparative investigation method. The data were used to evaluate the impact of the capital adequacy ratio on profitability in Nepalese microfinance institutions and to inform future research. A causal-comparative investigation design was applied to assess the effects of capital adequacy ratios on profitability in Nepalese microfinance institutions. The study followed a quantitative approach.

### 3.2 Population and sample, and sampling design

In Nepal, there are 57 registered MFIs as of May 2025. For this study, 10 microfinance institutions were purposively selected based on their significant presence, influence, and financial diversity. The sampling is structured to capture both wholesaler and retailer microfinance institutions, reflecting different operational scales and business models. Specifically, FMLBSL, SKBLBSL, and RSDCLBSL are categorized as wholesaler microfinance institutions, accounting for 30 percent of the total sample. These wholesalers primarily provide bulk loans to smaller microfinance institutions or cooperatives rather than directly to individual clients.

The remaining 70 percent of involves seven retail microfinance institutions, including CLBSL, NULBSL, FMLBSL, GBLBSL, MMLBSL, NMBLBSL, and NADEPLBSL. Those institutionss involve direct end customers, provided they are limited to entities and collections for investment decisions.

This sampling method safeguards a representative sample of large-scale, extensive creditors and smaller-scale, retail-oriented microfinance breadwinners, enabling a comprehensive and proportional examination of the capital adequacy ratio (CAR) and its impact on profitability across diverse administrative categories within Nepalese microfinance institutions.

### 3.3 Nature and sources of data

The main source of the data was secondary, comprising the annual reports of the selected microfinance institutions. They provided the thorough financial statements essential for analyzing capital adequacy and profitability. Furthermore, the research combined data and institutions, drawing on both published and unpublished sources. Applicable resources such as academic journals, newspapers, and magazines were studied to capture broader industrial tendencies and background context. Additionally, annual reports of concerned MFIs and and Nepal Rastra Bank NRB are important sources of consistent financial data and regulatory information.

### 3.4 Data collection instruments and procedures

The data collected from the annual reports of the selected institutions and from reports published by the Nepal Rastra Bank were organized and analyzed in Excel. The use of suitable data collection methods enhanced the credibility and value of the research outcomes.

### 3.5 Data analysis tools and methods

Financial and statistical techniques, such as mean, standard deviation, correlation, and multiple regression, were used to analyze data from the sampled institutions’ annual reports.

### 3.6 Financial tools

The financial ratios, such as return on assets, return on equity, capital adequacy ratio, and leverage ratio, size, GDP growth, inflation, and type, were included. This study employs panel regression to examine the impact of independent variables on the dependent variable. The specific regression model used in this study has been analyzed accordingly.

$$\text{Profitability}=\beta_{0}+\beta_{1}{\mathrm{CAR}}_{\mathrm{it}}+\beta_{2}{\mathrm{LR}}_{\mathrm{it}}+\beta_{3}{\text{SIZE}}_{\mathrm{it}}+\beta_{4}{\text{GDPgr}}_{\mathrm{it}}+\beta_{5}{\mathrm{INF}}_{\mathrm{it}}+\beta_{6}{\text{TYPE}}_{i}+\mu_{t}+\omega_{i}+\varepsilon_{\mathrm{it}}$$

Where,

Profitability is measured in the form of ROE (Return on equity) and ROA (Return on assets).

CAR = Capital adequacy ratio.

LR = Leverage ratio.

SIZE = Institutional size in terms of the log of total assets.

GDPgr = The growth rate of gross domestic product.

INF = Inflation rate.

TYPE = Types of microfinance institutions: 0 Retail and 1 Wholesale.

## 4. Results and discussion

In this study, return on assets and return on equity are dependent variables. Capital adequacy ratio, leverage ratio of the company, size, GDP growth, inflation and type of MFIs are independent variables.

### 4.1 Descriptive analysis

Table 1 presents descriptive statistics for the variables.

Table 1 examines 7 selected study variables from sampled institutions, with data collected over 100 observations (10 institutions × 10 years). The study variable, ROE, ranges from −1.88 to 4.48, with a mean of 1.63 and a standard deviation of 1.24. Similarly, ROA has a mean of 13.41 and a standard deviation of 8.71. It indicates that the sample institutions were achieving a satisfactory level of profitability. However, some institutions had the lowest return and demanded expanding their operations effectively and taking profitable steps.

**Table 1.** Descriptive statistics of the variables.

Variables	Minimum	Maximum	Mean	Std. Deviation
ROE	−1.88	4.48	1.63	1.24
ROA	1.37	38.67	13.41	8.71
CAR	2.73	35.00	12.68	4.38
LR	−54.67	1153.00	40.90	159.38
SIZE	6.66	10.89	9.25	0.93
GDPgr	−2.73	8.98	4.10	3.34
INF	2.78	8.79	5.81	1.86

Source: Annual reports of selected microfinance institutions.

The capital adequacy ratio ranges from 2.73 to 35, average ratio was 12.68, with a standard deviation of 4.38, indicating lower risk on the CAR, which is satisfactory. Likewise, leverage ratio whose minimum range was −54.66 and maximum range was 1153, whose average range was 40.90, and standard deviation was 159.38. The standard deviation reflected significant inequalities in the debt financing requirements; some sampled institutions were heavily leveraged, while others had minimal or negative leverage. The size of institutions in terms of total assests ranges from 6.66 to 10.89, indicates low deviation (0.93 standard deviation). In the case of GDP growth and Inflation, the level of deviation is relatively higher. It reveals that macroeconomic indicators are not stable. Sustainability of financial institutions largely depends on macroeconomic stability ( Svartzman et al., 2021).

### 4.2 Correlation analysis

The correlation analysis examined the relationship between the study variables. The variables were measured as positive or negative, effective or not. Table 2 indicates the correlation matrix.

**Table 2.** Correlation matrix.

	ROE	ROA	CAR	LR	SIZE	GDPgr	INF
**ROE**	1						
**ROA**	.907 ^**^	1					
**CAR**	0.168	0.143	1				
**LR**	−0.280	−0.140	−0.067	1			
**SIZE**	.288 ^**^	.247 ^*^	−0.028	−.231 ^*^	1		
**GDPgr**	0.160	0.143	0.038	0.098	−0.068	1	
**INF**	−0.024	−0.022	−0.101	−0.014	−0.149	−.441 ^**^	1

** Correlation is significant at the 0.01 level (2-tailed).

* Correlation is significant at the 0.05 level (2-tailed).

Source: Annual reports of selected microfinance institutions.

The strength and direction of the relationships between profitability indicators among explanatory variables are high ( Table 2). The findings indicate that ROA and ROE are significantly and positively correlated (r = 0.907, p < 0.01). It suggests that institutions are able to deliver substantial returns through their asset utilization. The CAR exhibits a weak, statistically insignificant positive association with ROA (r = 0.168, p > 0.05), as well as with ROE (r = 0.143, p > 0.05).

LR shows a negative association with both profitability events. Increased leverage reduces profitability and increases the risks associated with extreme debt requirements. Likewise, size of total assets is positively and significantly associated with profitability of MFIs. GDP growth has a positive relationship with profitability, whereas inflation has a negative association with the profitability of MFIs.

### 4.3 Panel unit root test

Running a panel unit root test before regression is critical because estimating models with non-stationary data leads to spurious regressions, invalid statistical inference, and incorrect model selection. Panel unit root test: Im-Pesaran-Shin unit-root test is presented in Table 3.

**Table 3.** Panel unit root test: Im-Pesaran-Shin unit-root test.

Variable	IPS Test (Level)		IPS Test (1st Diff )		OI
ROE	0.001	p > 0.05	0.000	p < 0.05	I(1)
ROA	0.261	p < 0.05			I(0)
CAR	0.043	p < 0.05			I(0)
LR	0.000	p < 0.05			I(0)
SIZE	0.001	p < 0.05			I(0)
GDPgr	0.000	p < 0.05			I(0)
INF	0.000	p < 0.05			I(0)

Note: IPS: Im-Pesaran-Shin; OI: Order of Integration.

Source: Annual reports of selected microfinance institutions.

In Table 3, Im-Pesaran-Shin (IPS) unit root test is evaluated at level for variables ROE, ROA, CAR, LR, SIZE, GDPgr, and INF. All variables except ROE are found stationary I(0). ROE is found stationary at first difference I(1).

### 4.4 Panel regression models

Fixed effects and random effects regression models were used under the panel regression model. The data are structured in a panel, and the study is intended to test the determinants of Nepalese microfinance institutions’ profitability. The Hausman test was used for choosing the model. Additional controls such as institutional size, GDP growth, inflation rate, and institutional type were included to examine the effects on profit. Models 1 and 2 under the panel regression indicate ROE and ROA, respectively.

Model 1: ROE

$${\text{ΔROE}}_{\mathrm{it}}=\beta_{0}+\beta_{1}{\mathrm{CAR}}_{\mathrm{it}}+\beta_{2}{\mathrm{LR}}_{\mathrm{it}}+\beta_{3}{\text{SIZE}}_{\mathrm{it}}+\beta_{4}{\text{GDPgr}}_{\mathrm{it}}+\beta_{5}{\mathrm{INF}}_{\mathrm{it}}+\beta_{6}{\text{TYPE}}_{i}+\mu_{t}+\omega_{i}+\varepsilon_{\mathrm{it}}$$

Model 2: ROA

$${\mathrm{ROA}}_{\mathrm{it}}=\beta_{0}+\beta_{1}{\mathrm{CAR}}_{\mathrm{it}}+\beta_{2}{\mathrm{LR}}_{\mathrm{it}}+\beta_{3}{\text{SIZE}}_{\mathrm{it}}+\beta_{4}{\text{GDPgr}}_{\mathrm{it}}+\beta_{5}{\mathrm{INF}}_{\mathrm{it}}+\beta_{6}{\text{TYPE}}_{i}+\mu_{t}+\omega_{i}+\varepsilon_{\mathrm{it}}$$

Tables 4 and 5 present the panel regression results of fixed and random effects as dependent variables of ROE and ROA, respectively.

**Table 4.** Panel regression results: ROE as a dependent variable.

Variables	FE Coef	FE SE	FE p_value	RE Coef	RE SE	RE p_value
Intercept				5.341	7.372	0.469
CAR	0.040	0.232	0.865	−0.030	0.155	0.848
LR	0.001	0.004	0.757	0.001	0.004	0.852
SIZE	−0.253	1.319	0.848	−0.472	0.686	0.492
GDPgr	0.109	0.193	0.574	0.108	0.183	0.555
INF	−0.332	0.368	0.370	−0.347	0.352	0.323
TYPE				1.197	1.512	0.430
Total Sum of Squares			2345.9			2451.5
Residual Sum of Squares			2271.6			2338.4
R-squared			0.032			0.046

Hausman test χ ^2^ = 0.367, df = 5, p = 0.996.

Source: Authors’ calculation based on a *nnual report of selected microfinance.*

In Table 4, none of the regression coefficients are statistically significant in Model 1: ROE in both the fixed-effects panel regression model and the random-effects regression model. Regression coefficients for SIZE and GDP growth in the fixed-effects panel regression model, and CAR, LR, SIZE, GDP growth, and TYPE are statistically significant in Model 2 ( Table 5). However, the fixed-effect model is preferred to the random-effect model as per the result of the Hausman test.

**Table 5.** Panel regression results: ROA as a dependent variable.

Variables	FE Coef	FE SE	FE p_value	RE Coef	RE SE	RE p_value
Intercept				−2.404	1.439	0.095
CAR	0.002	0.032	0.716	0.085	0.031	0.007
LR	0.000	0.001	0.683	−0.002	0.001	0.009
SIZE	−0.399	0.163	0.016	0.254	0.130	0.051
GDPgr	0.052	0.028	0.070	0.092	0.036	0.011
INF	−0.002	0.052	0.963	0.094	0.066	0.154
TYPE				−0.798	0.315	0.011
Total Sum of Squares			63.674			135.5
Residual Sum of Squares			55.381			104.6
R-squared			0.130			0.229

Hausman test χ ^2^ = 688.85, df = 5, p = 0.000.

Source: Authors’ calculation based on a *nnual reports of selected microfinance institutions.*

Instead of performing different analyses for Retail MFIs and Wholesale MFIs, a dummy variable is created to separate these two, which is also beneficial when samples are not sufficient. Since TYPE is time-invariant (constant over time for each MFI), it is automatically absorbed by the unobserved entity effect in the Fixed Effects (FE) estimator. It is directly estimated in the Random Effects (RE) model. Here, the regression coefficient for TYPE is statistically significant; the profitability of Retail MFIs and Wholesale MFIs is different.

### 4.5 Diagnostic tests

To validate the premises for the panel data regression, a number of diagnostic tests were performed. These diagnostics ensured that the models included were adequate to be utilized in investigating the determinants of capital adequacy with the profitability on microfinance institutions in Nepal. Table 6 indicates the diagnostic tests.

**Table 6.** Diagnostic tests.

Studentized Breusch-Pagan test for heteroskedasticity check		
Model	BP-statistic	p-value
Model 1: ROE	11.604	0.040
Model 2: ROA	10.508	0.062

Wooldridge test for serial correlation		
Model	F-statistic	p-value
Model 1: ROE	363.78	0.000
Model 2: ROA	6.38	0.013

Jarque-Bera normality test		
Model	JB-statistic	p-value
Model 1: ROE	143.8	0.000
Model 2: ROA	23.7	0.000

Source: Authors’ calculation based on the annual reports of selected microfinance institutions.

In Table 6, the panel heteroskedasticity (Modified Wald test), autocorrelation (Wooldridge test), and normality (Jarque-Bera test) confirm no issues of heteroskedasticity, autocorrelation and non-normality.

## 5. Discussion, conclusion, and policy implications

### 5.1 Discussion

This research explored the effect of capital adequacy on the profitability of Nepalese microfinance institutions. Capital adequacy shows a weak correlation with profitability. Leverage ratio exhibits a negative association indicating increased leverage reduces profitablity and raises the risks. Likewise, size of total assets positively and significantly associated with profitability of MFIs. GDP growth has positive relationship with profitability whereas inflation has a negative association with profitability of MFIs. These findings underscore the urgent need for a comprehensive method for capital structure, liquidity management, and debt operations to restore financial performance.

The inverse relationship between capital adequacy and profitability validates earlier findings. A high level of capital adequacy strengthens financial stability and constrains lending capacity and operational efficiency, thereby weakening profitability ( Ikpesu & Oke, 2025; Saeed & Donkoh, 2026). It implies that excessive capital adequacy increases opportunity costs, as funds allocated to capital are not accessible for income-generating undertakings ( Mamun et al., 2022). Huy et al. (2024) found that commercial banks have achieved their equity capital requirements through successful compliance with all safety standards which protect against credit risk exposure according to legal regulations.

The panel regression model observed that regression coefficients for SIZE and GDP growth in the fixed-effects panel regression model, and CAR, LR, SIZE, GDP growth, and TYPE are statistically significant for ROA. This finding is consistent with the Yadav et al. (2022) that the association of firm size, growth and profitability is estimated using dynamic panel fixed effects including firm-specific variables and macroeconomic as well as financial development factors to explain profitability. Leverage ratio indicates significant positive influences on profitability ( Shabani et al., 2019; Goet, 2022). Although this study did not find a statistically significant effect on ROE. Profitability of microfinance institutions is not only determined by the sum of capital and investments, but also through the strategic balance between financial stability and operational efficiency. High levels of capital adequacy necessitate restrained management to avoid diminishing returns. The regression coefficient for TYPE is statistically significant; the profitability of Retail MFIs and Wholesale MFIs is different. This finding aligns with the study made by Marr and Tubaro (2013) that type of MFIs is related to social and commercial interests.

Capital adequacy is critical for financial security; excessively high levels can reduce profitability. Microfinance institutions must balance capital cushions with lending and investment activities. Leverage must be used carefully, as excessive reliance on debt can intensify risk without offering improved returns. A deliberate approach to capital management, liquidity planning, and debt operations helps institutions achieve sustainable and efficient financial performance.

### 5.2 Conclusion

The capital structure, asset size, and leverage are the determinants of the profitability of microfinance institutions. One interesting result is that the type of microfinance institution is statistically significant; the profitability of retail MFIs and wholesale MFIs is different. It indicates that wholesale MFIs are more capitalized while less profitable than retail MFIs. This implies that capital position does not necessarily explain into higher profitability. Retail business models in terms of financial performance may not necessarily work in wholesale business models. The impact of capital position on profitability depends on the business model to a certain extent. The unobserved factors, which include portfolio quality and operating efficiency, explain nearly all variations in profitability. Capital adequacy is a significant determinant of the type of MFI profitability in Nepal.

### 5.3 Policy implications

The effect of capital adequacy on profitability is contingent on the institutional business model. Policymakers should not apply a one-size-fits-all regulation but develop diverse supervisory and capital adequacy frameworks for wholesale and retail MFIs. The institutional business model approach enables microfinance institutions to achieve profitability and financial resilience and ultimately promote sustainable financial inclusion in Nepal. This study concentrates on a small sample of 10 Nepalese MFIs over 10 years, mainly in regard to capital adequacy and a specific set of firm-specific and macroeconomic determinants of profitability. Future research could extend the sample size, add other governance and risk factors, and conduct a rural-urban analysis. This study is particularly useful for regulators and microfinance institutions for policy formulation.

## Ethical approval

Ethical approval is not required because the data are secondary.

## Data availability statement

### Underlying data

Repository name: Impact of capital adequacy on the profitability of microfinance institutions in Nepal. https://doi.org/10.5281/zenodo.18860868 ( Lamichhane et al., 2026).

The project contains the following underlying data:

• Data used for the research.docx (Data used for the research - Appendix: Years/Variables, ROA, ROE, CCR, SCR, CAR, CRR, LR, Company)

## Extended data

There is no extended data.

Data are available under the terms of the Creative Commons Attribution 4.0 International license “No rights reserved” data waiver (CC-BY 4.0).
