# Supplementary material for: Capital Adequacy as a Driver of Profitability: Panel Evidence from Nepalese Wholesale and Retail Microfinance Institutions
Source: F1000Res. 2026 Sep 29;15:447. Originally published 2026 Mar 27. [Version 3] doi: 10.12688/f1000research.178815.3 (PMC13594598; doi:10.12688/f1000research.178815.3)
Supplement: Peer review history

# Reviewer report: Reviewer response for version 2

**DOI:** 10.5256/f1000research.201893.r510153

**Published:** 2026-09-22

**Authors:** Lilian Gumbo

**Affiliations:** 1 University of South Africa, Pretoria, South Africa

The research is well structured and well-articulated. The authors explained the background and problem statement comprehensively.

 The study primarily examined the dependent and independent variables associated with earnings, liquidity, and capital. However, it overlooked an important control variable, size, which is relevant to the model. The size of a financial institution, measured as the natural logarithm of its assets, plays a critical role in capital adequacy models.

 The literature and theory on banks treat size as an important variable in capital adequacy and liquidity models. The first two papers cited by the authors in the empirical literature included size as an important variable in the regression model. The omission of a relevant variable generally leads to model specification error; additionally, the determination of capital is closely associated with the size and nature of risk-weighted assets. Hence, the importance of including size as an important variable in the model to be consistent with literature and theory.

****

 The choice of 10 micro financial institution out of the 57 should be statistically justified using sample size statistical methods since the study adopted a quantitative research approach. The microfinance sector in Nepal seems very diverse; an explanation of the variability of the selected microfinance institutions in representing the complete set of microfinance institutions is also vital, for example in terms of ownership, deposit-taking or non-deposit-taking (size was considered in selecting the sample, as explained by wholesale and retail microfinance institutions.

 The authors should consider adding the random error term in their model to ensure completeness in model presentation (this does not affect results since data analysis software automatically applies it).

Is the work clearly and accurately presented and does it cite the current literature?

Yes

If applicable, is the statistical analysis and its interpretation appropriate?

Yes

Are all the source data underlying the results available to ensure full reproducibility?

Yes

Is the study design appropriate and is the work technically sound?

Yes

Are the conclusions drawn adequately supported by the results?

Yes

Are sufficient details of methods and analysis provided to allow replication by others?

Yes

Reviewer Expertise:

NA

I confirm that I have read this submission and believe that I have an appropriate level of expertise to confirm that it is of an acceptable scientific standard.

# Reviewer report: Reviewer response for version 2

**DOI:** 10.5256/f1000research.201893.r497295

**Published:** 2026-08-14

**Authors:** Margaret Kosgei, Dr Vincent Shiundu

**Affiliations:** 1 Kenyatta University, Nairobi, Kenya

Abstract: The research concludes that ‘’regulators and policymakers may focus on improving the financial and non-financial performance of MFIs’’, yet the study’s focus was on financial performance as measured by ROA and ROE and not on non-financial performance

 1: The title reads ‘Impact of Capital Adequacy on the Profitability of Microfinance Institutions in Nepal’. The independent variable has been conceptualized to have 5 constructs raising the 5 specific objectives. However, one of the 5 objectives is the capital adequacy ratio, the independent variable construct itself.

 Whereas CAR is indicated to consists of two main components: Tier 1 capital, which includes essential equity and disclosed capital, and Tier 2 capital, which includes subordinated debt and mixed instruments, it is not clear which was adopted for the research

  

1.
  - The problem can be supported by bringing in statistics or trends on profitability of the MFIs based on ROA and ROE

- The objectives stated herein are three; one on current status, relationship and impact. Clarify on the focus of the research study

 2. The objectives stated in section 2 deviates from the focus of the study as envisaged in the research hypothesis, data analysis and discussion of findings.

 3. 1: The author can beef up the theory write up by brining out the application, criticism and relevance to the study. The introduction section mentioned capital structure theories and none was include in the theoretical review.

 5.2 The Author needed to have two correlation outputs; one for ROA and the other for ROE against the five independent variables. As it has ben done on the regressions

 Table 10: Does not include the output of   χ ^2^ = 8.947 with a p-value of 0.030 to justify choice of the fixed effects (FE) model. 

 Table 11: Does not include the output of χ ^2^ equal to 6.723 and p value of 0.081 to justify the use of the random effects (RE) model 

 6.2 The conclusion deviates from the main study objective which was focussing on the impact of capital adequacy ratio on profitability. It now concludes on ‘’the capital structure, assets, and leverage on the profitability of Nepalese microfinance institutions is affected by core capital, supplementary capital, capital adequacy, the cash reserve ratio, and the leverage ratio’’

 In general, there is need for consistency in research objectives

Is the work clearly and accurately presented and does it cite the current literature?

Yes

If applicable, is the statistical analysis and its interpretation appropriate?

Partly

Are all the source data underlying the results available to ensure full reproducibility?

Yes

Is the study design appropriate and is the work technically sound?

Partly

Are the conclusions drawn adequately supported by the results?

No

Are sufficient details of methods and analysis provided to allow replication by others?

Partly

Reviewer Expertise:

Finance

We confirm that we have read this submission and believe that we have an appropriate level of expertise to confirm that it is of an acceptable scientific standard, however we have significant reservations, as outlined above.

# Reviewer report: Reviewer response for version 2

**DOI:** 10.5256/f1000research.201893.r489130

**Published:** 2026-07-01

**Authors:** Olcay Ölçen

**Affiliations:** 1 Istanbul Gelisim University, Istanbul, Turkey

I am satisfied with changes.

Is the work clearly and accurately presented and does it cite the current literature?

Partly

If applicable, is the statistical analysis and its interpretation appropriate?

No

Are all the source data underlying the results available to ensure full reproducibility?

Partly

Is the study design appropriate and is the work technically sound?

No

Are the conclusions drawn adequately supported by the results?

Yes

Are sufficient details of methods and analysis provided to allow replication by others?

No

Reviewer Expertise:

NA

I confirm that I have read this submission and believe that I have an appropriate level of expertise to confirm that it is of an acceptable scientific standard.

# Reviewer report: Reviewer response for version 2

**DOI:** 10.5256/f1000research.201893.r489129

**Published:** 2026-06-08

**Authors:** Quoc-Huy Nguyen

**Affiliations:** 1 Lac Hong University, Biên Hòa, Vietnam

- In the regression equation, the authors simultaneously included Core Capital Ratio (CCR), Supplementary Capital Ratio (SCR), and Capital Adequacy Ratio (CAR) as independent variables. From a financial perspective, CAR is essentially the sum of Tier 1 capital (equivalent to CCR) and Tier 2 capital (equivalent to SCR). Including both the components and their sum in the same model inevitably leads to perfect or near-perfect multicollinearity. The correlation matrix in Table 3 itself demonstrates a very high correlation between CAR and CCR (r=0.697). Although in the latter part of the paper (Table 12), the authors removed CCR and SCR to retain only CAR, presenting a series of models and tables (from Table 4 to Table 9) based on a model with severe multicollinearity is unreasonable and diminishes the integrity of the scientific paper.

 - The dataset utilized is panel data, comprising 10 microfinance institutions observed over a 10-year period, yielding 100 observations. However, the initial results (Tables 4 through 9) appear to employ the Pooled Ordinary Least Squares (Pooled OLS) method. Ignoring the unique, time-invariant characteristics of each institution over time in a Pooled OLS model leads to biased results. Furthermore, the absence of pre-regression statistical diagnostic tests (such as tests for normality, heteroskedasticity, autocorrelation, and unit roots) compromises the reliability of the estimates.

 - The research framework (Figure 1) and the regression models are completely devoid of control variables. The results indicate an Adjusted R-square of only 0.279 for the dependent variable ROA and 0.264 for ROE. This implies that approximately 70% of the variance in profitability remains unexplained by the current model. To meet standard econometric benchmarks, the authors must incorporate basic control variables such as institutional size (Log of Total Assets), growth rate, or macroeconomic factors (GDP growth, inflation), which are highly critical in the Nepalese market. The authors themselves acknowledged this deficiency in the conclusion section of the paper.

 - In the literature review and methodology sections, the authors emphasize filling the research gap by distinguishing between wholesale (30% of the sample) and retail (70% of the sample) microfinance institutions. Nevertheless, in the entirety of the quantitative analysis, the data from these two groups are aggregated without any differentiation. To properly address the stated gap, the authors should have utilized a dummy variable (e.g., 1 for wholesale, 0 for retail) or conducted separate regressions to compare the impact of capital adequacy on these specific business models.

Is the work clearly and accurately presented and does it cite the current literature?

Partly

If applicable, is the statistical analysis and its interpretation appropriate?

No

Are all the source data underlying the results available to ensure full reproducibility?

Partly

Is the study design appropriate and is the work technically sound?

Partly

Are the conclusions drawn adequately supported by the results?

No

Are sufficient details of methods and analysis provided to allow replication by others?

Partly

Reviewer Expertise:

Financial Economics; Development Economics; Sustainable Development

I confirm that I have read this submission and believe that I have an appropriate level of expertise to state that I do not consider it to be of an acceptable scientific standard, for reasons outlined above.

# Response

**Published:** 2026-09-15

**Authors:** Bharat Ram Dhungana

Dear Reviewer(s),

 Thank you for the opportunity to submit a revised draft of the manuscript, “Impact of Capital Adequacy on the Profitability of Microfinance Institutions in Nepal”. We have revised our title based on our research findings, and now our research paper title is *Capital Adequacy as a Driver of Profitability: Panel Evidence from Nepalese Wholesale and Retail Microfinance Institutions*. We appreciate the time and effort you and the reviewers dedicated to providing feedback on our manuscript, and we are grateful for the insightful comments and valuable improvements to our paper. We have incorporated all the reviewers' suggestions to the fullest extent. We are grateful to the anonymous reviewers of our manuscript. Please find below the response sheet for the reviewers' and the editor's perusal.  We have submitted the revised manuscript after incorporating all valuable and insightful feedback.

 Thank you.

**Comment 1: **In the regression equation, the authors simultaneously included Core Capital Ratio (CCR), Supplementary Capital Ratio (SCR), and Capital Adequacy Ratio (CAR) as independent variables. From a financial perspective, CAR is essentially the sum of Tier 1 capital (equivalent to CCR) and Tier 2 capital (equivalent to SCR). Including both the components and their sum in the same model inevitably leads to perfect or near-perfect multicollinearity. The correlation matrix in Table 3 itself demonstrates a very high correlation between CAR and CCR (r=0.697). Although in the latter part of the paper (Table 12), the authors removed CCR and SCR to retain only CAR, presenting a series of models and tables (from Table 4 to Table 9) based on a model with severe multicollinearity is unreasonable and diminishes the integrity of the scientific paper.

**Author(s) response to comment 1: **Thank you for your valuable feedback. We are now happy with your suggestion and have modified the model. We have drastically revised the paper, addressed all issues, and incorporated them.

*Profitability=* *β* *0* *+* *β* *1*CAR *it* *+* *β* *2*LR *it* *+* *β* *3*SIZE *it* *+* *β* *4*GDPgr *it* *+* *β* *5*INF *it* *+* *β* *6*TYPE *i* *+* *µ* *t* *+* *ω* *i* *+* *ε* *it*

 Where,

 Profitability is measured in the form of ROE (Return on equity) and ROA (Return on assets)

 CAR = Capital adequacy ratio

 LR = Leverage ratio

 SIZE = Institutional size in terms of the log of total assets

 GDPgr = The growth rate of gross domestic product

 INF= Inflation rate

 TYPE = Types of microfinance institutions: 0 Retail and 1 Wholesale

 We appreciate your rigorous review.

**Comment 2: **The dataset utilized is panel data, comprising 10 microfinance institutions observed over a 10-year period, yielding 100 observations. However, the initial results (Tables 4 through 9) appear to employ the Pooled Ordinary Least Squares (Pooled OLS) method. Ignoring the unique, time-invariant characteristics of each institution over time in a Pooled OLS model leads to biased results. Furthermore, the absence of pre-regression statistical diagnostic tests (such as tests for normality, heteroskedasticity, autocorrelation, and unit roots) compromises the reliability of the estimates.

**Author(s) response to comment 2: **Thank you for your remarkable suggestions.

 We have addressed them.

**Model 1: ROE**

 ΔROE *it* *=* *β* *0* *+* *β* *1*CAR *it* *+* *β* *2*LR *it* *+* *β* *3*SIZE *it* *+* *β* *4*GDPgr *it* *+* *β* *5*INF *it* *+* *β* *6*TYPE *i* *+* *µ* *t* *+* *ω* *i* *+* *ε* *it*

**Model 2: ROA**

 ROA *it* *=* *β* *0* *+* *β* *1*CAR *it* *+* *β* *2*LR *it* *+* *β* *3*SIZE *it* *+* *β* *4*GDPgr *it* *+* *β* *5*INF *it* *+* *β* *6*TYPE *i* *+* *µ* *t* *+* *ω* *i* *+* *ε* *it*

 As per your suggestion, we have used both FE and RE models together with the Hausman test, according to the results presented above.

**Comment 3: **The research framework (Figure 1) and the regression models are completely devoid of control variables. The results indicate an adjusted R-square of only 0.279 for the dependent variable ROA and 0.264 for ROE. This implies that approximately 70% of the variance in profitability remains unexplained by the current model. To meet standard econometric benchmarks, the authors must incorporate basic control variables such as institutional size (Log of Total Assets), growth rate, or macroeconomic factors (GDP growth, inflation), which are highly critical in the Nepalese market. The authors themselves acknowledged this deficiency in the conclusion section of the paper.

**Author(s) response to comment 3: **Thank you for your valuable feedback. We agree with your suggestion. We added variables as per your feedback and developed a new model.

*Profitability=* *β* *0* *+* *β* *1*CAR *it* *+* *β* *2*LR *it* *+* *β* *3*SIZE *it* *+* *β* *4*GDPgr *it* *+* *β* *5*INF *it* *+* *β* *6*TYPE *i* *+* *µ* *t* *+* *ω* *i* *+* *ε* *it*

**Comment 4: **In the literature review and methodology sections, the authors emphasize filling the research gap by distinguishing between wholesale (30% of the sample) and retail (70% of the sample) microfinance institutions. Nevertheless, in the entirety of the quantitative analysis, the data from these two groups are aggregated without any differentiation. To properly address the stated gap, the authors should have utilized a dummy variable (e.g., 1 for wholesale, 0 for retail) or conducted separate regressions to compare the impact of capital adequacy on these specific business models.

**Author(s) response to comment 4: **Thank you for your valuable feedback. We agree with your suggestion.

 We again collected data, added the TYPE variable (Type of MFIs, i.e., wholesale and retail), and analyzed it. Our main findings are related to the type of MFIs. As per our findings, we also modified our title.

 We are grateful to you for your insightful comments.

# Reviewer report: Reviewer response for version 1

**DOI:** 10.5256/f1000research.197251.r480748

**Published:** 2026-05-05

**Authors:** Olcay Ölçen

**Affiliations:** 1 Istanbul Gelisim University, Istanbul, Turkey

The research concentrates on the development of microfinance institutions, considering Capital Adequacy and other profitability measures. Although the research plan, section and sub-section designs were developed and presented well,

1. The research suffers from consistency, validity and reliability problems in the research period, sampling and methodology (regression and ANOVA) or description of them. For instance, you should offer some statistical tests before regression analysis, such as Normality, heteroskedasticity, autocorrelation, unit roots, multicollinearity, etc.
2. The research needs rigorous proofreading and elimination of mistakes in important places, as in the research period.
3. The research has restrictions and limitations because of the research period, the number of Nepalese Microfinance Institutions (Micro), and the place of Nepal in the developing world (Macro). 

I offer not approved status

Is the work clearly and accurately presented and does it cite the current literature?

Partly

If applicable, is the statistical analysis and its interpretation appropriate?

No

Are all the source data underlying the results available to ensure full reproducibility?

Partly

Is the study design appropriate and is the work technically sound?

No

Are the conclusions drawn adequately supported by the results?

Yes

Are sufficient details of methods and analysis provided to allow replication by others?

No

Reviewer Expertise:

Finance and financial modelling

I confirm that I have read this submission and believe that I have an appropriate level of expertise to state that I do not consider it to be of an acceptable scientific standard, for reasons outlined above.

# Reviewer report: Reviewer response for version 1

**DOI:** 10.5256/f1000research.197251.r476171

**Published:** 2026-04-24

**Authors:** Quoc-Huy Nguyen

**Affiliations:** 1 Lac Hong University, Biên Hòa, Vietnam

- In the study's regression equation:

 ROA = a + b1 x CCR + b2 x SCR + b3 x CAR + b4 x CRR + b5 x LR

 The authors include Core Capital Ratio (CCR), Supplementary Capital Ratio (SCR), and Capital Adequacy Ratio (CAR) as independent variables. Financially, CAR is essentially the sum of Tier 1 capital (equivalent to CCR) and Tier 2 capital (equivalent to SCR). Including both the components and their sum in the same model will inevitably lead to perfect or near-perfect multicollinearity.

 Evidence: The correlation matrix in Table 3 confirms a very high correlation between CAR and CCR (r = 0.697).

 => The authors should conduct diagnostic tests for multicollinearity, such as the Variance Inflation Factor (VIF). It is recommended to establish two separate models: one using only CAR, and another breaking it down into CCR and SCR to avoid biasing the regression coefficients.

 - The dataset consists of 10 microfinance institutions observed over a 10-year period (from 2014/15 to 2023/24). This constitutes a panel dataset with 100 observations. However, the authors appear to use standard Pooled Ordinary Least Squares (Pooled OLS) regression, as indicated by the standard ANOVA and Coefficients tables.

 => For panel data, ignoring the unique characteristics of each institution over time leads to biased results. The authors should apply and compare the Fixed Effects Model (FEM) and the Random Effects Model (REM), subsequently using the Hausman test to select the most appropriate model.

 - Standard econometric models in banking and finance research require control variables to avoid omitted variable bias. In the research framework (Figure 1), control variables are entirely absent.

 => The results show an Adjusted R-square of only 0.279 for ROA and 0.264 for ROE. This implies that approximately 70% of the variation in profitability remains unexplained. The authors should incorporate basic control variables, such as institutional size (Log of Total Assets), growth rate, or macroeconomic factors (GDP growth, inflation), which are critical in the Nepalese market.

 - The authors emphasize a significant research gap: the distinction between wholesaler microfinance institutions (30% of the sample) and retailers (70% of the sample). However, in the quantitative analysis, the data for these two groups is aggregated without any differentiation.

 => To address the stated gap, the authors should include a dummy variable (e.g., 1 for wholesalers, 0 for retailers) in the regression model or perform separate regression analyses for each group to compare whether the impact of capital adequacy on profitability differs between these business models.

 - In Section 5.1 (Descriptive analysis), there is a significant error stating that data was collected *"over 100 years"* instead of accurately stating "100 observations" (10 institutions x 10 years). This must be corrected immediately to avoid misleading readers.

 - The mean ROE reported in the text is "134108," whereas the table shows 13.4108. A decimal point was omitted in the text.

 References:

 https://doi.org/10.47738/jads.v5i1.156

 Huy, N. Q., and L. Q.Dinh. 2025. “Balancing Bank Profits With Sustainable Development Goals: Examining the Pivotal Role of Financial Stability.” Sustainable Development33, no. S1: 1182–1199. https://doi.org/10.1002/sd.70057.

Is the work clearly and accurately presented and does it cite the current literature?

Partly

If applicable, is the statistical analysis and its interpretation appropriate?

No

Are all the source data underlying the results available to ensure full reproducibility?

Partly

Is the study design appropriate and is the work technically sound?

Partly

Are the conclusions drawn adequately supported by the results?

No

Are sufficient details of methods and analysis provided to allow replication by others?

Partly

Reviewer Expertise:

Financial Economics; Development Economics; Sustainable Development

I confirm that I have read this submission and believe that I have an appropriate level of expertise to state that I do not consider it to be of an acceptable scientific standard, for reasons outlined above.

## References

[1] : Applied Data Science for Analyzing the Mediating Role of Digital Transformation Influencing Banking Business Efficiency in Vietnam. *Journal of Applied Data Sciences* .2025;6(3) : 10.47738/jads.v6i3.8072031-2045 10.47738/jads.v6i3.807
